# Multi-Allelic Major Effect Genes Interact with Minor Effect QTLs to Control Adaptive Color Pattern Variation in *Heliconius erato*

**DOI:** 10.1371/journal.pone.0057033

**Published:** 2013-03-22

**Authors:** Riccardo Papa, Durrell D. Kapan, Brian A. Counterman, Karla Maldonado, Daniel P. Lindstrom, Robert D. Reed, H. Frederik Nijhout, Tomas Hrbek, W. Owen McMillan

**Affiliations:** 1 Department of Biology and Center for Applied Tropical Ecology and Conservation, University of Puerto Rico, Rio Piedras, Puerto Rico; 2 Department of Entomology and Center for Comparative Genomics, California Academy of Sciences, San Francisco, California, United States of America; 3 Center for Conservation and Research Training, Pacific Biosciences Research Center, University of Hawaii at Manoa, Honolulu, Hawaii, United States of America; 4 Department of Biological Sciences, Mississippi State University, Mississippi State, Mississippi, United States of America; 5 Department of Biology, University of Guam, Mangilao, Guam; 6 Department of Ecology and Evolutionary Biology, Cornell University, Ithaca, New York, United States of America; 7 Department of Biology, Duke University, Durham, North Carolina, United States of America; 8 Biology Department, Institute of Biological Sciences, Federal University of Amazonas, Manaus, AM, Brazil; 9 Smithsonian Tropical Research Institute, Panama City, Panama; Oxford Brookes University, United Kingdom

## Abstract

Recent studies indicate that relatively few genomic regions are repeatedly involved in the evolution of *Heliconius* butterfly wing patterns. Although this work demonstrates a number of cases where homologous loci underlie both convergent and divergent wing pattern change among different *Heliconius* species, it is still unclear exactly how many loci underlie pattern variation across the genus. To address this question for *Heliconius erato*, we created fifteen independent crosses utilizing the four most distinct color pattern races and analyzed color pattern segregation across a total of 1271 F2 and backcross offspring. Additionally, we used the most variable brood, an F2 cross between *H. himera* and the east Ecuadorian *H. erato notabilis*, to perform a quantitative genetic analysis of color pattern variation and produce a detailed map of the loci likely involved in the *H. erato* color pattern radiation. Using AFLP and gene based markers, we show that fewer major genes than previously envisioned control the color pattern variation in *H. erato*. We describe for the first time the genetic architecture of *H. erato* wing color pattern by assessing quantitative variation in addition to traditional linkage mapping. In particular, our data suggest three genomic intervals modulate the bulk of the observed variation in color. Furthermore, we also identify several modifier loci of moderate effect size that contribute to the quantitative wing pattern variation. Our results are consistent with the two-step model for the evolution of mimetic wing patterns in *Heliconius* and support a growing body of empirical data demonstrating the importance of major effect loci in adaptive change.

## Introduction

Butterflies are among the most charismatic insects and their kaleidoscopic color variation has fascinated scientists for centuries. The wing pattern radiations of *Heliconius* butterflies in particular have become a useful model for understanding the evolution, genetic basis, and development of complex adaptive variation [1]–[6]. The genus *Heliconius* is incredibly diverse and characterized by repeated cases of extreme divergence and convergence in wing patterns. While the first can occur between closely related species or races within species, the latter is the product of Müllerian mimicry between distantly related species. As an example, the two unpalatable co-mimetic species *H. erato* and *H. melpomene* diverged from each other more than 8 million years ago [7], [8] and do not interbreed, yet they share identical wing patterns. With greater than 25 parapatric races, each with a distinctive warning-color pattern, *H. erato* and *H. melpomene* represent one of the best example of a parallel radiation [9]–[11]. This patchwork of wing color-pattern races within a species and convergent mimetic matching between *H. erato* and *H. melpomene* provides a vivid example of the importance of natural selection in shaping phenotypic variation [12], [13]. Although recent studies points to a few large-effect loci controlling major phenotypic changes within both species [1], [14], [15], we still have a limited understanding of the overall number and effect sizes of the loci that control these complex and highly polymorphic color-patterns.

The work of Turner and Crane [16] and Sheppard *et al.* [11] created the foundation to explore the genetic architecture of wing pattern variation in *Heliconius erato* and *Heliconius melpomene*. Together these studies described 22 distinct color pattern genes in *H. erato* and 17 in *H. melpomene* based on observations of how color pattern variation segregated across a series of opportunistic crosses. The relationship among some of the genes described by Sheppard and collaborators [11] has been subject to molecular analysis over past few years. One of the most remarkable finding to emerge from this work is the observation that the same genomic intervals appear to underlie both pattern convergence between distantly related species and pattern divergence between closely related species [14], [17], [18]. Recent work has succeeded in positional cloning two distinct color pattern genes and to show that each gene, underlies a diversity of wing phenotypes across the genus [1], [19]. Despite these advances in understanding the genetic basis of *Heliconius* wing pattern, the work of Sheppard *et al.* [11] remains the only attempt to provide a comprehensive understanding of the genetic determinants of wing pattern variation across the two co-radiations. However, the independent characteristics of each cross, the inability to compare results with a unified genetic map, the small-brood sizes and other limits of their crossing design leaves a number of unanswered questions. In particular, it still is not clear how many independent genes underlie the color pattern radiation. Moreover, we know little about the distribution of their phenotypic effects and interactions to produce color patterns. More recently, studies on the genetic architecture of *Heliconius* wing color pattern variation focused on mapping in order to describe the action of single genes action in stand-alone crosses [20]–[23] or looked at genes homology between different species [3], [14]. Thus, researchers have not yet described the variation seen across an entire adaptive radiation.

To fill these gaps we used a combination of genetic mapping and quantitative genetic analysis to uncover the genetic architecture of inter-racial variation in *H. erato* wing patterns. Our strategy allows us to dissect the genetic control of wing color pattern variation in *H. erato* in order to easily understand the homology of major color pattern genes between distinct morphs of *H. erato* and to characterize the contribution of alternative loci to the total quantitative variation of major patterns variants. We generated a comprehensive description of the genetic architecture of *H. erato* wing color pattern diversity in three major steps. First, we created large replicate mapping families of four distinctive races of *H. erato* by crossing each race to the inter-fertile sister species *H. himera* [22], [23]. Second, using a high-resolution amplified fragment length polymorphism (AFLP) screen, we identified several markers tightly linked to the major color pattern loci modulating pattern and mapped these markers across our collection of pedigrees to produce a single “reference” linkage map of the *H. erato* color pattern radiation. Third, we conducted an initial quantitative analysis of color pattern variation segregating in one of our crosses to more deeply explore the underlying architecture of pattern variation. Our high definition linkage mapping and a quantitative trait locus (QTL) analysis allows us 1) to explore the phenotypic effects of major patterning loci, 2) to gain a more detailed appreciation for the genetic architecture of pattern variation across the *H. erato* radiation, and 3) to identify other loci that underlie color variation and determine their overall contribution to the quantitative variation observed in our crosses.

Overall, our results paradoxically show that while the genetic basis of major wing pattern elements variation between races is simpler than previously envisioned, these elements are affected by quantitative variation that is more complex than ever depicted. Several color pattern genes previously assigned to distinct chromosomes are actually located in the same genomic region, suggesting that they are most likely allelic variants of a single locus. Nonetheless, we uncovered new loci that modify these color patterns and explain a substantial component of phenotypic variation segregating in our crosses. Thus, with our experimental design it was possible to test hypotheses regarding the number and effect size of major versus minor loci underlying the adaptive wing pattern diversity in *H. erato*. Finally, our findings reinforce past models about the evolution of new color patterns within species and add to the growing body of empirical work demonstrating the importance of major effect loci in adaptive evolution.

## Results

Overall, our collection of crosses represents most of the major wing pattern phenotypes found in nature. A total of fifteen mapping families, including several replicated broods of each of the four different races of *H. erato* crossed to *H. himera*, were generated for this study (see Table S1a). These crosses provide the foundation for a comprehensive assessment of color pattern variation, integrative linkage analysis of the major color pattern genes, and an initial QTL study of pattern variation. The data suggest that the genetic architecture of wing color pattern variation in *H. erato* is controlled by few major gene and multiple addictive loci of smaller effect.

### Qualitative assessment of wing color pattern variation

**Figure 1:**
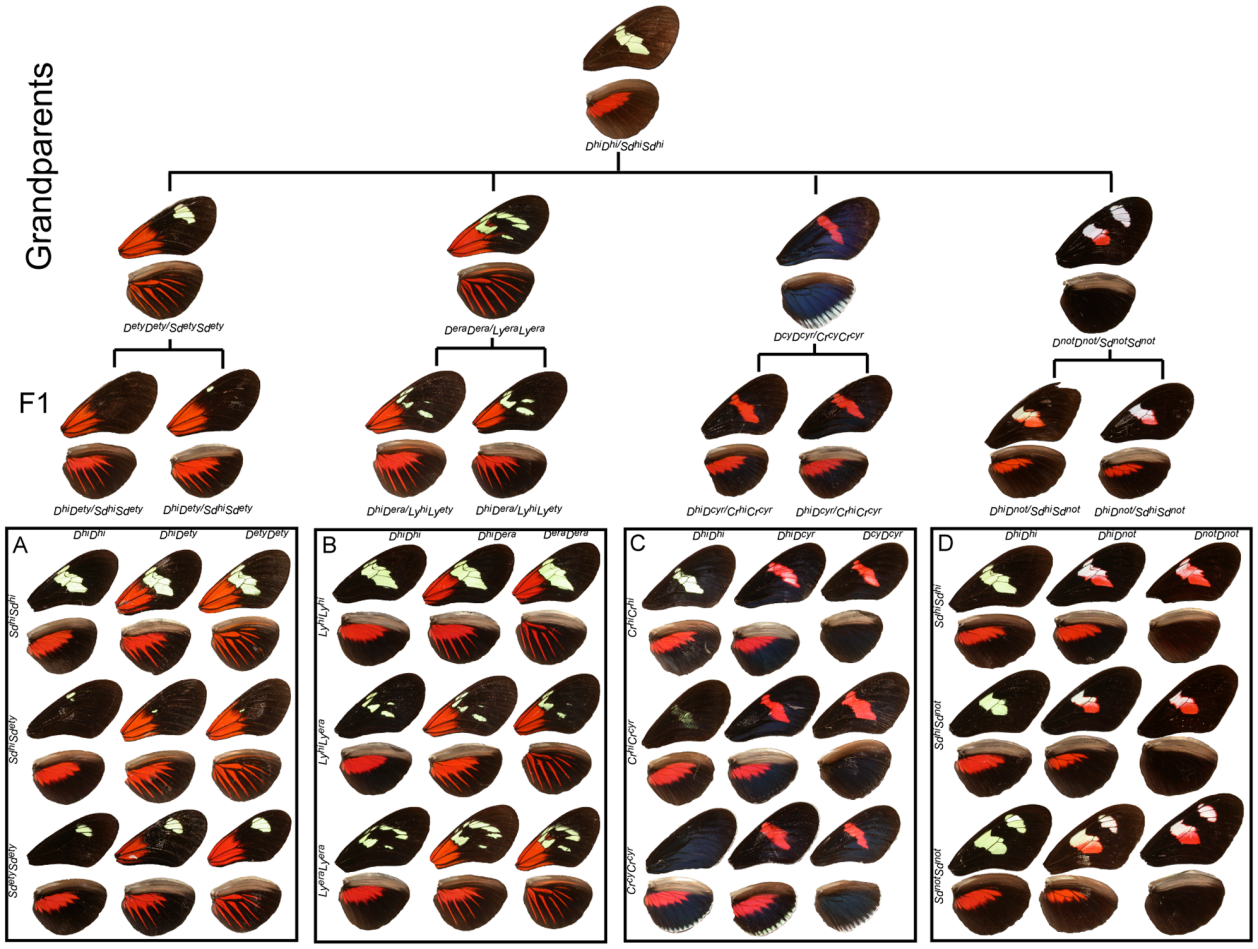
Crossing strategy and color pattern variation. Mendelian segregation of the major color pattern genes in *H. erato* as seen in our collection of crosses. Four distinct geographic races (from left *H. e. etylus* (A), *H. e. erato* (B), *H. e. cyrbia* (C), and *H. e. notabilis* (D)) were crossed to *H. himera* (top) to generate backcross (BC) and F2 mapping families. The nine major phenotypes produced by the segregation of alternative alleles at major loci are arranged in each box with *D* (top) and *Sd*, *Ly*, *Sd*, and *Cr* (left) with contributions from *H. himera* (top and left) and *H. erato* (bottom and right). Heterozygotes for these major color pattern loci are found in the middle column and row with double heterozygotes in the center. Inferred genotypes are indicated across the top and on the side of each box (see text for details).

**Figure 2:**
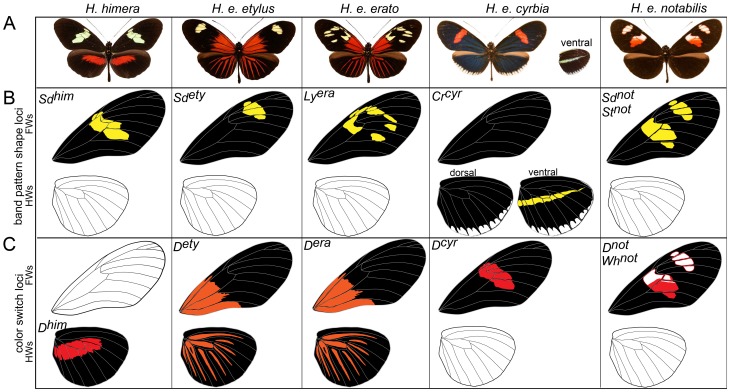
Major effect alleles at major color pattern loci segregating in the *H. himera*×*H. erato* mapping families. (A) Distinct geographic races of *H. erato* and the sister species *H. himera* used in the mapping crosses. (B) Homozygous phenotypes of the loci that control the distribution of black pigment (“melanin shutter genes”). (C) Homozygous phenotypes produced by the *D* locus. Allelic variation at this locus controls the distribution of red and yellow color patterns. See text for brief description of major color pattern loci.

**Figure 3:**
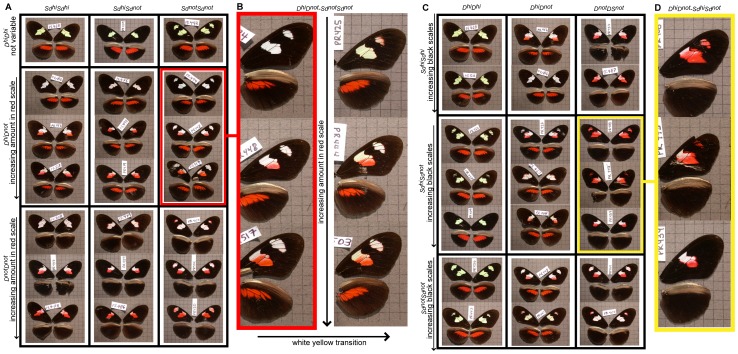
Variation of color and forewing band shape in a *H. himera×H. erato notabalis* F2 cross. In Panel A, the variation of white/red scale proportions in the forewing is presented across particular color pattern genotypes. All the offspring homozygous with the *H. himera D* allele showed only yellow pigments, whereas individuals homozygous for the *H. e. notabilis* alleles possessed were white. Heterozygous individuals were typically white, although there was some variation (Panel B). Panel C and D shows variation in forewing band shape among F2 individuals as a function of *Sd* genotype.

Major wing pattern variation is largely explained by simple bi-allelic inheritance at a handful of loci of large effect in general agreement with previous studies [11], [20], [22], [24]. Overall, natural variation of the size and shape of the bands and bars elements in the fore- and hindwing were consistent with the concept that regions of black scales genetically define the position and size of these elements [25]–[27]. This idea is most easily demonstrated by the forewing band patterns of F1 individuals. In all our crosses, the F1 phenotypes are highly predictable and can be created by the superposition of parental black patterns (Figure 1). This interpretation is very different from the original interpretation of Sheppard *et al.* [11] who viewed color patterns as being expressed on a black background. Red color elements were also easily understood by allelic variation at a single large effect locus. The phenotypic effects of major loci in specific crosses (Figures 1 and 2) are summarized below:

*H. himera×H. e. etylus*: Allellic variation at two codominant loci largely explains phenotypic variation in our *H. himera*×*H. e. etylus* crosses [11], [20], [22], [24]; see also Figure 1A and Figure 2). The *D* locus controlled all red and orange variation on both forewings and hindwings. Red and orange pattern elements were codominant and both F1 parents had intermediate phenotypes featuring *H. erato* derived orange patches at the base of the forewing, and orange hindwing rays blended with red hindwing bands derived from *H. himera*. In the F2 offspring, the orange basal forewings and hindwings rays were always inherited together in our crosses and qualitative variation in forewing band size and shape was consistent with bi-allellic inheritance at a single locus [22]. Variation in the size and shape of the forewing band (Figure 3) was controlled by the *Sd* locus [22], which operates by adding patches of melanin in the middle part of the forewing. The almost complete absence of any yellow scales in the forewings of the F1 was due to the activity of alternative *Sd* alleles that complement each other and induces melaninization either proximally or distally (Figure 3)

*H. himera×H. e. erato*: Variation in these crosses segregated in a way similar to the *H. himera*×*H. e. etylus* crosses. Specifically, we attributed most of the phenotypic variation to allelic variation at two loci, one affecting the presence of red and orange (*D*) in the forewing and hindwing and the other affecting the size and shape of the forewing band. The gene controlling the broken band phenotype of *H. e. erato* was previously called *Ly* by Sheppard *et al.* [11] (see also Figure 1B and Figure 2). Variation of the orange *H. erato* basal forewing and hindwing rays and the red *H. himera* hindwing bar resembled exactly the segregation observed in the *H. himera×H. e. etylus* cross for both F1 parents and F2 offspring. Variation in the size and shape of the forewing band again suggested the interaction of alternative complementary melanin alleles. The parental F1 had an intermediate phenotype with a reduced yellow pattern. More precisely there were five yellow spots specific to the parental F1s with their position defined by wing veins: 1) the discal spot, 2) a spot between Cu1b and Cu1a wing veins, 3) a spot between Cu1a and M3, 4) a spot between Sc and R1 wing veins, and 5) a spot between R1 and R2 wing veins (Figure 1B and 2). Although the yellow spots in the F1 perfectly reflected the heterozygote intermediate *himera/erato* phenotype, the forewing band size and shape variation became more complex in the F2 offspring. In particular, the extent that the forewing band extended towards the apex of the wing was highly variable, suggesting the presence of additional modifier loci (RP and WOM, personal observation).

*H. himera×H. erato cyrbia*: Most of the phenotypic variation in our *H. himera*×*H. e. cyrbia* crosses could be explained by two major color pattern genes, *Cr* and *D*, [14], [20], [23] (see also Figure 1C Figure and 2). Red was controlled by the *D* locus and F1 individuals had red on both the fore and hindwing. In addition a second locus, *Cr*, was responsible for the yellow ventral line and white fringes of the *H. e. cyrbia* hindwing and interacted with *D* to determine the presence/absence of the forewing band. Similar to *Sd* above, the *Cr* locus largely acted by controlling melanic patches on both the forewing and the hindwing, with *H. e. cyrbia Cr* alleles positioning a large melanic patch across the middle of the forewing and portions of the hindwing; whereas the *Cr* allele in *H. himera* put melanin across the entire hindwing. The epistatic interaction between the *Cr^hi^* and *D^cyr^* alleles created some scoring difficulties for *Cr*. When individuals were homozygous for the *H. himera D* allele, the forewing band was either completely absent (*Cr^cyr^Cr^cyr^*), nearly completely covered with melanic scales (*Cr^cyr^Cr^hi^*), or fully yellow (*Cr^hi^Cr^hi^* ^)^. In contrast individuals heterozygous for *D* had a red forewing band [20]. There was evidence for additional variation in forewing band size and shape and for variation in the intensity of iridescence (RP and WOM, personal observation), but this variation was not easily scored qualitatively.

*H. himera×H. e. notabilis*: Qualitative variation in individuals from *H. himera×H. e. notabilis* mapping families followed a simple segregation pattern consistent with bi-allelic variation at two major effect loci (Figure 1D and Figure 2). These broods also showed the most quantitative phenotypic variation among our collection of crosses (Figure 1D and Figure 3). The F1 parents of these crosses presented an intermediate phenotype with the red hindwing bar of *H. himera* and a partial red forewing band of *H. e. notabilis* (Figure 1C and Figure 2), presumably representing alternative alleles at the red locus (*D*). However, the amount of red present in the forewing band varied extensively in the F2, from nearly fully red to red restricted to the margins of the forewing bands. In addition, yellow pigments were always present in F2 individuals that had the *H. himera* red hindwing bar, whereas, individuals with red on both the forewing and hindwing, or on the forewing only, had white scale cells in the forewing bands. The geometric shape of the forewing band in our *H. himera* and *H. e. notabilis* crosses was complicated as well. Previous crossing experiments implicated three loci, *St*, *Sd* and *Round* (*Ro*), as responsible for the distinctive split forewing band of *H. e. notabilis* [11]. In our cross, the F1 individuals had an intermediate forewing band between *H. himera* and *H. e. notabilis*, with black melanin scales phenotypically masking yellow or white scales. However, there were typically some remnants of the upper forewing band in F1 individuals, represented by diffuse pigmentation around the edge of the middle forewing band near the intersection of Cu1a – M3 and M3 – M2 wing veins (Figure 1). F2 individuals showed variable expression of the upper forewing patch (Figure 3D).

### Mapping across *H. erato* color pattern radiation

We examined 23 AFLP primer combinations (*EcoCN/MseCNN*) in several of our *H. erato* reference crosses. Using this strategy we identified a number of AFLP markers tightly linked (1–3 cM) to the main color pattern genes (*Sd^ety^*, *Sd^not^*, *St^not^*, *D*, and *Ly*) segregating in our crosses, and used them as landmarks for cross- comparison of linkage between *H. erato* races and for BAC library screening [15], [28]. For the *Cr* color pattern gene we utilized a gene-based marker linked to *Cr* color pattern gene (“*GerTra”* - *Rab geranylgeranyl transferase beta subunit*, *βggt-II*) [14], which was successfully amplified and mapped in the *F2-Not9* [15].

**Figure 4:**
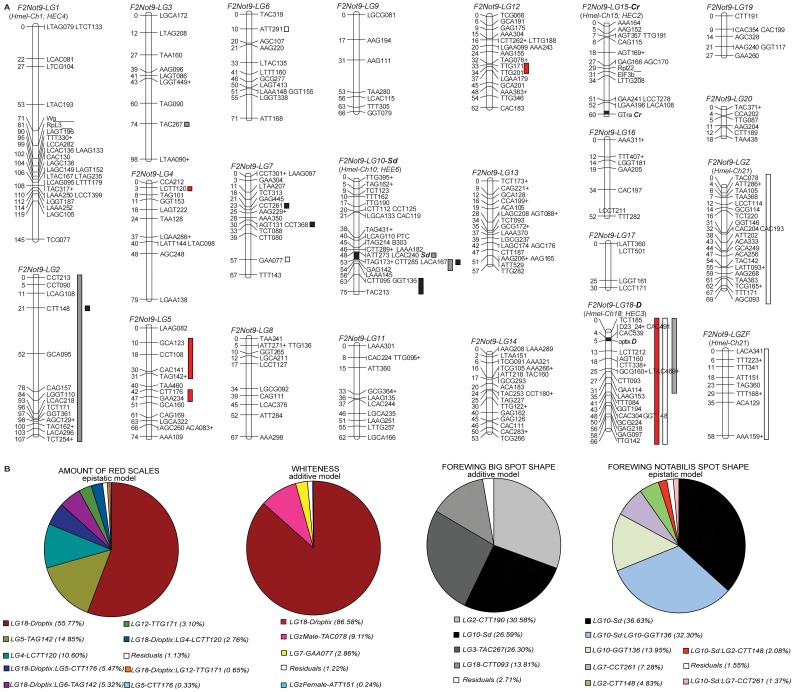
*F2-Not9* linkage map and overall QTL analysis. (A) *F2-Not9* linkage map and overall QTL analysis of the *H. himera×H. erato notabilis* cross showing the 20 autosomal and two sex linkage groups generated with AFLP and co-dominant anchor loci (Tables S5, Table S6, Table S7, and Table S8). Numbers of the left side represent distance in cM rounded to the closest integer value, while letters on the right side represent marker names. Approximate positions of major color pattern genes (*Sd*-LG 10, *D*-LG 18, *Cr*-LG 15) are indicated with a black square within each linkage group. Vertical bars next to the chromosomes represent QTL regions with colors corresponding to phenotypes measured: red bars = redness; white bars = whiteness; grey bars = Big-Spot (BS); and black bars = Not-Spot (NS). (B) Pie charts show the relative contributions of individual markers to the total variance explained when all significant QTLs were analyzed under the best model (additive or epistatic). Note that *F2-Not9* LG 1, LG 10, LG 18 and LG 15 correspond to LG 4, LG 3, LG 6 and LG 2 in Kapan *et al.* (2006) and to LG 1, LG 10, LG 18 and LG 15 in Jiggins *et al.* (2005) respectively. Linkage analysis and autosomal LG numbers for the *F2-NotF29* reference map are arranged with the same numbers as *H. melpomene* when homology could have ben established.

To generate a high resolution map of wing color pattern loci across these *H. erato* races we examined almost 1500 cumulative AFLP polymorphisms from three independent *H. erato×H. himera* crosses (*F2-Not9*. *F2-Et2* and *F2-He11*). In the *F2-Not9* cross alone, which was used as the backbone reference linkage map (Figure 4A), we scored 604 AFLP markers representing 245 male informative (MI), 213 backcross informative (BI), and 146 female informative (FI) respectively. After filtering the male and backcross informative AFLP markers for missing genotypes, undetectable phase, and segregation distortion (*G* test, *P*<0.05) 20 AFLP loci (5 MI and 16 BI) were discarded and did not enter the final dataset to construct the *F2-Not9* linkage map (Figure 4A). In addition to these AFLP loci, we also mapped nine gene-based markers (Table S2). Roughly half of the total AFLP markers scored in our crosses were *H. himera* alleles. The segregation of *H. himera* alleles in the offspring of our crosses increased the chance to identify co-segregating markers shared between independent crosses of *H. erato* (Table S3), and thus the possibility to use them as anchor loci to align the independent linkage analysis and test for color pattern gene homology (Figure 4A). Utilizing gene based markers and major color pattern genes as anchor loci we were also able to compare previous linkage maps in *H. erato* [22] and *H. melpomene* [21], which allowed cross-study assignment of linkage groups. As a result, we identified four linkage groups LG 1, LG 10, LG 18 and LG 15 from our analysis that were previously named LG 4, LG 3, LG 6 and LG 2 in *H. e. etylus* [22] (Figure 4A). For these linkage groups we used the chromosome numbers utilized in *H. melpomene* [21], [29] since it represent the first fully sequenced and reference genome among *Heliconius* butterflies. The remaining linkage groups were ordered by decreasing size in centimorgans (cM)

Taking advantage of the absence of crossing over in female Lepidoptera [30], [31], we identified ‘chromosome prints’ for the 20 autosomal chromosomes and sex chromosomes using female informative (FI) markers grouped at LOD 8.0 [22]. We also used unexpected discrepancies among FI AFLP markers [21], [22], given the lack of recombination in females, to infer the experiment-wide error rate, which was only ≈1% [15]. Amongst the female-informative markers, there were three markers that grouped at >LOD8.0 with sex that were positive only in females, thus identifying the W chromosome (WF-CC-CAC-182, WF-CT-CAG-140, WF-CT-CCT-153). We identified the sex (Z) chromosome from 8 FI markers present in males only. This and the autosomal chromosome prints obtained with FI markers were then used to build the final genetic map by assigning the linkage group for BI markers [21], [22] and using BI and MI markers grouped at LOD 4.0 or higher. Interestingly, we identified 12 BI markers grouping with the Z chromosome print. These 12 markers formed two non-mergeable linkage groups at LOD 4.0 with MI markers. These markers were genotyped in many of the same individuals, eliminating the usual cause for non-merged maps, [32] highlighting the possibility of divergence between *H. e. notabalis* and *H. himera* Z chromosomes in this study. Finally, our final mapped dataset consisted of 446 loci, 197 censored BI loci [21], [22], 240 MI loci and 9 gene based markers. Out of the total 437 AFLP loci, 171 markers generated 47 haplotypes and therefore reduced the unique breaks in the final genetic map to 313 (47 haplotypes and 266 single markers). Given this level of coverage we identified the maximum likelihood interval containing the *D* and *Sd* loci to be around 3 cM and 5 cM respectively. If we assume a one-to-one correspondence between physical and recombination size [22], [23] the above interval can be translated to 0.8 Mb and ∼1.4 Mb for the *D* and *Sd* color pattern genes respectively.

### *D* and *Wh* map to the same region of the genome

**Figure 5:**
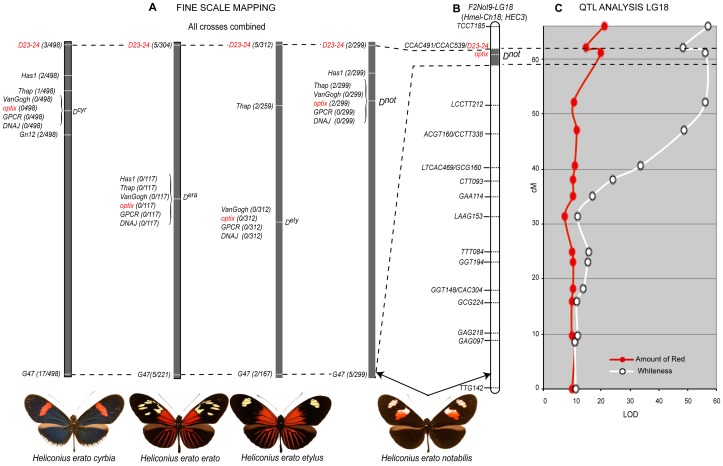
The homologous position of the *D* color pattern gene in four different races of *H. erato* and its pleiotropic effect on forewing color pattern. (A) The fine resolution map (over 600 Kb) of several co-dominant markers linked to the *D* color pattern gene across all our collection of crosses. The relative position and number of recombinant individuals for the eight co-dominant markers linked to the *D* color pattern gene (see Table S1) confirmed the homologous position of *D* across all *H. erato* races used in our crosses. The numbers within brackets in panel A represent the total number of recombinant individuals generated by combining the information from all crosses (Table S1). (B) The linkage analysis of LG 18 in the *F2-Not9* cross and the relative position of the *D* locus on the chromosome, including the position and name of AFLP loci, two of the eight co-dominant markers (*D23/24* and *optix*) and the *D* locus along the chromosome. (C) QTL analysis for the forewing band color (red and/or white) on LG 18. The very high LOD score coupled with the significance of the *D* locus make the entire LG 18 a significant QTL (*P*<0.01) (black stars) (see Table S5).

We characterized five AFLP loci, all in the *F2-Not9* cross, tightly linked to *D* (Table S4). The *D*-linked marker CAC491 also segregated in the *F2-Et2* cross and was important for positional cloning the *D* red locus [15], [28]. Using a suite of nine co-dominant markers tightly linked to CAC491 [28], we showed *D* is responsible for all red pattern variation across our collection of *H. erato* crosses (Figure 5A).

Previous researchers [11] speculated that a distinct locus, *White* (*Wh*), unlinked to the *D* locus, controlled the presence of white scales in the forewing of *H. e. notabilis*. However, in our *H. himera* by *H. e. notabilis* crosses white and yellow color variation perfectly co-segregated in the two homozygote types: white pigment was never present in individuals that were homozygous for the *H. himera D* allele, whereas, all the individuals homozygous for the *H. e. notabilis D* allele showed white scales on their forewings (Figure 3). Heterozygous individuals had mainly white scales in the forewing bands, with the exception of 12 individuals that appeared to have a mix of white and yellow scales. QTL analysis confirmed this pattern and a QTL centered at the *D* locus largely explained the switch between white and yellow scale cell type in the forewing band (Figure 5C and Table 1). The LOD score around the *D* locus was so strong compared to any other QTLs that it resulted in the entire linkage group being significant (P<0.01; Figure 5C and Figure 4A). We also observed a second QTL for the last marker at end of LG18, which we believe most likely to be erroneous since it is known that chromosome ends might introduce false positives [33], [34].

**Table 1:** Summary of QTL analysis of forewing color and band size.

*AMOUNT OF RED*						*WHITE - YELLOW SWITCH*					
Locus		Contribution		Significance		Locus		Contribution		Significance	
Marker	LG	% QTL Variation		Pr(>F)		Marker	LG	% QTL Variation		Pr(>F)	
D/optix	18	42.05%		9.25E-010	***	D/optix	18	67.26%		2.00E-016	***
TAG142	5	22.00%		0.0001337	***	TAC078	ZM	18.13%		0.0008947	***
CTT176	5	16.23%		0.002187	***	ATT151	ZF	15.99%		0.002434	**
TTG171	13	18.12%		0.002537	***	GAA077	7	13.97%		0.00609	**
LCTT120	4	18.49%		0.002152	***						
**All additive**		67.20%				**All additive**		76.69%			
**All epistasis**		88.37%		0.002122		**All epistasis**		83.31%		0.6221	

Additive model						Additive model					
Marker	Df	% QTL Variation	F value	Significance Pr (>F)		Marker	Df	% QTL Variation	F value	Significance Pr (>F)	
D/optix	3	64.44%	29.4842	2.21E-012	***	D/optix	3	86.58%	71.1699	2.00E-016	***
TAG142	3	17.16%	7.853	0.0001395	***	TAC078	3	9.11%	7.4914	0.0001908	***
LCTT120	4	12.25%	5.6045	0.0005763	***	ATT151	2	0.24%	0.1951	0.8232032	
TTG171	4	3.59%	1.6406	0.1739443		GAA077	3	2.86%	2.3487	0.0794119	
CTT176	2	0.38%	0.1753	0.839545		Residuals	74	1.22%			
Residuals	69	2.19%									

Epistatic model						Epistatic model					
Marker	Df	% QTL Variation	F value	Significance Pr (>F)		Marker	Df	% QTL Variation	F value	Significance Pr (>F)	
D/optix	3	55.77%	49.4012	1.12E-013	***	D/optix	3	83.97%	68.5034	2.00E-016	***
TAG142	3	14.85%	13.1578	3.70E-006	***	TAC078	3	8.84%	7.2107	0.0003994	***
LCTT120	4	10.60%	9.3904	1.75E-005	***	ATT151	2	0.23%	0.1878	0.829387	
TTG171	4	3.10%	2.7488	0.040922	*	GAA077	3	2.77%	2.2607	0.0925132	
CTT176	2	0.33%	0.2938	0.746994		D:TAC078	9	0.80%	0.6519	0.7474648	
D:TAG142	8	5.32%	4.7147	0.000379	***	D:ATT151	5	2.02%	1.6439	0.1653122	
D:LCTT120	9	2.76%	2.4424	0.025041	*	D:GAA077	9	0.84%	0.6824	0.7211298	
D:TTG171	9	0.65%	0.5793	0.80615		Residuals	41	0.54%			
D:CTT176	2	5.47%	4.8489	0.012877	*						
Residuals	41	1.13%									

*FOREWING BIG SPOT SHAPE*						*FOREWING NOTABILIS SPOT SHAPE*					
Locus		Contribution		Significance		Locus		Contribution		Significance	
Marker	LG	% QTL Variation		Pr(>F)		Marker	LG	% QTL Variation		Pr(>F)	
TAC267	3	19.26%		0.001518	**	GGT136	10	51.52%		1.12E-005	***
CTT093	18	18.34%		0.0008098	***	Sd	10	44.42%		0.0001139	***
CCT090	2	17.94%		0.0009795	***	CTT148	2	32.63%		0.002885	**
Sd	10	15.54%		0.002998	**	CCT261	7	30.66%		0.004662	**
**All additive**		61.98%				**All additive**		70.40%			
**All epistasis**		74.88%		0.661		**All epistasis**		86.80%		0.005249	

Additive model						Additive model					
Marker	Df	% QTL Variation	F value	Significance Pr (>F)		Marker	Df	% QTL Variation	F value	Significance Pr (>F)	
CCT090	3	30.58%	11.2814	3.76E-006	***	Sd	3	56.00%	13.5049	1.43E-005	***
Sd	3	26.59%	9.8095	1.67E-005	***	GGT136	2	21.33%	5.1432	0.01281	*
TAC267	4	26.30%	9.6997	2.48E-006	***	CCT261	3	11.14%	2.6854	0.0665	
CTT093	3	13.81%	5.0957	0.002965	**	CTT148	3	7.39%	1.7817	0.17441	
Residuals	72	2.71%				Residuals	27	4.15%			

Epistatic model						Epistatic model					
Marker	Df	% QTL Variation	F value	Significance Pr (>F)		Marker	Df	% QTL Variation	F value	Significance Pr (>F)	
CCT090	3	28.45%	10.6737	2.02E-005	***	Sd	3	36.63%	23.5622	6.40E-007	***
Sd	3	24.73%	9.2811	6.83E-005	***	GGT136	2	13.95%	8.9734	0.0015253	**
TAC267	4	24.46%	9.1772	1.65E-005	***	CTT148	3	4.83%	3.1086	0.0483064	*
CTT093	3	12.85%	4.8212	0.005391	**	CCT261	3	7.28%	4.6852	0.0117206	*
Sd:CTT093	9	2.86%	1.0725	0.401062		Sd:GGT136	1	32.30%	20.7819	0.0001709	***
Sd:CCT090	9	2.34%	0.8769	0.55247		Sd:CTT148	2	2.08&	1.3373	0.2840037	
Sd:TAC267	9	1.65%	0.6196	0.773936		Sd:CCT261	3	1.37%	0.8836	0.4656215	

The percent variation explained by individual QTLs when each QTL region was analyzed separately (Main Markers Contribution), when all significant QTLs were analyzed together assuming additivity of markers (additive model), and when all QTLs were analyzed together but assuming interactions between major effect loci and other QTLs (epistatic model) are presented for the forewing color and band size quantitative variation. For each QTL analyses [redness, white-yellow switch, Big-Spot shape (BS), and Not-Spot (NS)] the probability and the amount of variation explained by each model are shown. The relative contributions of particular QTLs under both additive and epistatic models for different aspects of forewing band color and size/variation are also detailed. Significance at the P = 0.05 (*), P = 0.01 (**), and P = 0.001 (***) levels for each locus is reported. Epistatic interaction between two distinct loci is reported with each locus ID separated by a colon.

### Evidence of a major forewing shutter gene: *St*, *Sd*, and *Ly* map to the same genomic region across *H. erato* races

**Figure 6:**
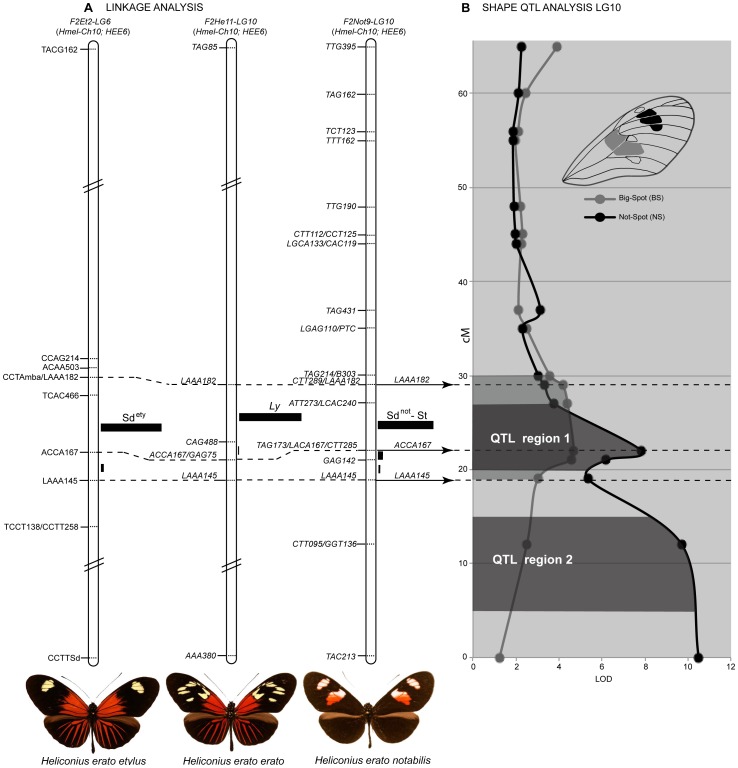
A single locus controls variation in the forewing black patterns of the *H. erato* radiation. (A) Linkage analysis of forewing black pattern variation segregating in three types of *H. himera*×*H. erato* F2 crosses. The dotted lines connect anchor AFLP loci indicating that the loci responsible for much of the variation in forewing band shape maps to homologous genomic intervals. The linkage analysis for the best placement of *Sd^ety^* was published in Kapan et al. (2006) while the genetic map of the other three loci *Ly*, *Sd^not^* and *St* is novel to this study. For each linkage analysis the black bars represent the probability of placement of each gene in a particular interval (see Kapan *et al.* 2006), while on the right side of the figure the overall probability for the best placement assuming a single locus affects these melanic patterns in all three crosses. (B) The effect of LG 10 on quantitative variation size of the lower (BS = grey line) and upper (NS = black line) forewing band is shown. For the lower band, there is evidence for a single QTL centered at *Sd* (*WntA*) that explains near 1/3 of the variation in the size of the band (QTL region 1). For the upper band, epistatic interaction between possibly two linked QTLs (QTL region 1 and QTL region 2) on LG 10 explain over 82% of the observed variation (see Table 1 and Table S8).

A number of distinct loci have been described to explain some aspect of forewing band shape variation in *H. erato* [11]. To determine if there was evidence for multiple loci segregating in our crosses between *H. himera* and different races of *H. erato*, we identified AFLP fragments tightly linked to the qualitative variation in forewing band shape across our collection of *H. himera*×*H. erato* crosses. Among these, *AAA145*, *ACA167* and *AAA182* were tightly linked to variation in forewing band shape in the *F2-Not9*, *F2-He11*, and *F2-Et2* mapping families. All three AFLP loci co-segregating in the three F2 crosses were excised and sequenced. Nucleotide sequences were nearly identical across the different color pattern races suggesting homology (Table S3) and these loci were used as anchors to align the three independent linkage analyses. Remarkably, variation in forewing band shape among the different *H. erato* races mapped to the same relative position of LG 10 (see Figure 6), suggesting that the same genomic interval was responsible for the majority of the forewing band variation observed across *H. erato* races. Moreover, the relative distance between the three AFLP fragments was almost identical in the different mapping families: a) distance between *LAAA145* and *ACCA167* was 3 cM in *F2-Et2*, 2.2 cM in *F2-He11*, and 2.9 cM in *F2-Not9*; while between *ACCA167* and *LAAA182* the relative distance was 8.8 cM in *F2-Et2*, 7.5 cM in *F2-He11*, and 7.1 cM in *F2-Not9*. The most likely placement of the three color pattern genes in each case was between *ACCA167* and *LAAA182* – an interval of ∼2 Mb assuming 276 kb/cM [22]. Given this observation, we have collapsed the following *Sd*, *St*, and *Ly*, which were previously thought to map to independent chromosomes or to very distant portion of the same linkage group, into a single relatively small region of the same chromosome. Although this region could easily contain hundreds of genes [15], in the light of recent studies [1], [3], [14], we speculate that our result most likely suggests a single forewing gene, which we call *Sd* for continuity with previous studies. *Sd* controls all wing color pattern variation in the middle of the forewing.

**Figure 7:**
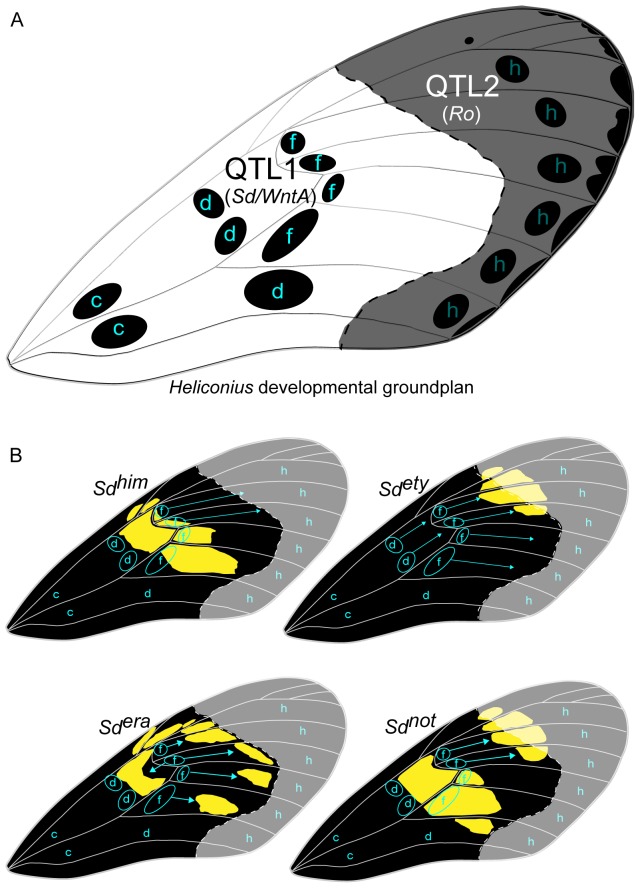
A developmental model for the observed phenotypic effects of the alleles at the *Sd* locus on the black pattern of the forewing. (A) The arrangement of organizing centers for *Heliconius* wing patterns. Lower case letters indicate serially homologous organizing centers (Nijhout and Wray 1988). The organizing centers control development of the black parts of the color pattern. The dotted line, delimit a hypothetical region (shaded) that represent s the influence of the two QTLs (*Sd = WntA* and possibly *Ro* color pattern genes) that control the upper forewing *H. e. notabilis* patch (see Figure 6). The *Sd* black melanization pattern is centered on the d and f NGP elements while the *Ro* melanization pattern coincide with the h NGP elements. (B) Expansion of the black pattern controlled by each of the *Sd* alleles. Each allele controls a different aspect of pattern expansion from organizing centers d and f. The *Sd^era^* allele also controls melanization along the wing veins, which breaks up the colored background pattern into discrete patches. The hypothetical region of action for the *Sd* and *Ro* color pattern genes is shown as well for the individual *H. erato* races.

Quantitative analysis of the forewing band variation in our reference cross *F2-Not9* (*H. himera*×*H. e. notabilis*) was largely explained by the *Sd* locus. QTL analysis of *H. notabilis* band size variation where measured in the large central area of the forewing band characterized proximally by a Big-Spot (BS) and distally by a Not-Spot (NS) (see Figure S1). These measures captures the complex shape changes of one versus two forewing patches and confirmed the major contribution of the *Sd* locus on LG 10 for both spots of the forewing (Figure 6 and Figure 4). However while the analysis on BS showed a single QTL region centered at the *Sd* locus (Figure 6: QTL region 1), the results for NS displayed two major QTL regions (Figure 6: QTL region 1 and QTL region 2), one of which matches *Sd*. The second QTL, more towards the bottom end of the LG 10 suggests the presence of an additional locus to delimit the size and shape of the upper forewing band. We hypothesized that this locus could be *Ro*, which has been described as the color pattern gene affecting the distal portion of the upper forewing band (Not-Spot = NS) [11], [35] (see also Figure 7).

Finally, the *Cr* locus, which segregates in our *H. himera*×*H. e. cyrbia* crosses, was unlinked to *Sd* and falls on LG 15 on our color pattern linkage map (Figure 4A). Mechanistically, this locus acts similar to *Sd* by altering the distribution of melanic scale cells. In *H. erato*, it seems to be most important on the hindwing and was responsible for the yellow dorsal and ventral hindwing bar and white fringes. Nonetheless, the *H. e. cyrbia Cr* allele acted across the middle of the forewing band similar to the action of *Sd*.

### QTL analysis identifies previously uncharacterized modifier loci

Despite the existence of major effect genes, a significant part of the variation observed in our broods had more complicated genetic control, which suggests the contribution of additional genes that may interact with the major effect loci. In order to understand the overall number and effect loci contributing to *Heliconius erato* wing color pattern variation, we performed a quantitative analysis on one of our *H. himera×H. e. notabilis* mapping families. Among our collection of crosses we chose the *F2-Not9* cross, because it presented some important advantages compared to the others: a) the most quantitative wing pattern variation, b) the segregation of variation previously ascribed to at least five color pattern genes (*D, Sd, St, Ro, Wh*), and c) the availability of a very high resolution genetic map. Two aspects to the wing color pattern variation in the *F2-Not9* cross emerged from our analysis. First, there was ample variation in the amount of red in the forewing band (Figure 3A and Figure 3B). Our quantitative analysis identified four additional QTLs on different linkage groups (one on LG 4, two on LG 5, and one on LG 12) that helped explain this variation (Figure 4A, Table 1 and Table S5). When all QTLs were considered simultaneously, an epistatic model was supported over a purely additive model (*p* = 0.002) and explained 88.3% of the variation (Table 1). After *D*, the next two strongest QTLs, one on LG 5 and another on LG 4, respectively explained 15% and 10% of the variation in the amount of red under an epistatic model (Table 1). Second, we similarly found evidence that other regions of the genome interact with *D* in order to switch between yellow and white scale cell types (Figure 5C, Table 1, and Table S6). A QTL centered in *D* controls most of the white – yellow variation (Figure 4 and Table 1); however, there was a QTL of moderate effect size on the Z chromosome, which explains over 10% of the variation. In this case, the effect was purely additive, together explaining about 75% of the variation in white – yellow scale types (Figure 1B and Table 1).

There was also substantial variation in the size and shape of the forewing bands in offspring of our reference *H. himera*×*H. e. notabilis* F2 brood (Figure 3C and 3B). QTL analysis of the relative size of the large central area of the forewing band (Big-Spot = BS), and the upper band (Not-Spot = NS) indicates the presence of a number of moderate sized (i.e. explaining more than 5% of variation) QTLs across the genome (Figure 4 and Table 1, Table S7 and Table S8). Many of these QTLs were quite large and explained as much variation in the relative size of the forewing band as the major *Sd* locus. For example, the variation in BS was controlled by four QTLs on four different linkage groups, including one on the chromosome containing the *D* locus (Figure 4), which acted largely additively to explain 63% variation in the size of this band (Table 1). Similarly, QTLs on three different chromosomes explained the variation in the upper *H. e. notabilis* forewing band (NS; see Figure 4A). In this case, QTLs interacted epistatically to explain over 95% of the variation in the size of the HS2 band. Much of this variation was explained by a strong epistatic interaction between the major melanin pattern gene (*Sd*) on LG 10 and another QTL on the same chromosome. The two QTL peaks were close together (see Figure 4A) and may reflect the action of a single large effect QTL. These QTLs, together with the interaction between them, explained about 82% of the variation in band size we observed in our crosses. In addition, there was evidence for moderate effect size QTLs on LG 2 and LG 7. Somewhat surprisingly, there was no evidence for any QTL near the *Cr*, which affected the same region of the forewing band in our *H. himera*×H. *e. cyrbia* cross.

## Discussion

The primary focus in this study was two-fold: (1) improve our understanding of the phenotypic control of major wing color pattern loci across races in order to test homology among these loci and (2) asses the quantitative genetic control of the remaining continuous variation. By combining linkage analyses in multiple broods with a single cross QTL study, we have developed a comprehensive foundation of the genetic architecture controlling *H.erato* wing color pattern variation. Integrating linkage analysis of major color pattern genes across multiple crosses of divergent color pattern forms confirms that pattern variation in *H. erato* is controlled by relatively few major effect loci that cause dramatic changes in pattern and color. Red, yellow, and white color variation is largely modulated by a large QTL centered on *optix*, which could represent a single locus (Figure 5, Table 1 and Table S6). Similarly, we demonstrate that most of the variation in forewing band shape and size, previously speculated to be controlled by several distinct loci, is in fact regulated by a single genomic region and probably by a single large effect gene (*WntA*), which we refer to as *Sd* (Figure 6, Table 1, Table S7 and Table S8). However, we cannot completely exclude the possibility that those genomic hotspots of butterflies wing color pattern adaptation centered on *optix* and *WntA* might represent several tightly linked loci. Although these loci have the largest effect, a significant component of the wing color pattern variation in *H. erato* is modulated by loci of smaller effect size. This is particularly true for the locus causing changes in the forewing band size and shape, which shows a significant qualitative and quantitative variation in all of our crosses and the greater number of “modifier” loci from our QTL analysis.

### Color variation in *Heliconius erato*

The yellow, orange, and red wing scales that compose the major wing pattern elements in *Heliconius* are colored by pigments derived from the tryptophan-ommochrome biosynthetic pathway [36]–[38]. Orange and red colors are ommochrome pigments synthesized in the developing scales cells, while the yellow pigment 3-hydroxykynurenine (3-OHK) circulates in the hemolymph and is taken up into scales cells shortly before adult emergence [36]. White scales appear to simply lack any color pigment [26], [27], [35]. White, yellow, orange, and red scales mature in a coordinated manner and share very similar structures. In contrast, melanic black scales develop much later and have distinct cuticle structures compared to non-black scales [39] (Reed pers. obs.). The correlation of developmental timing and cuticle structure between white, 3-OHK, and ommochrome scales implies that they may share a common specification mechanism. This hypothesis is consistent with our finding that in *H. erato* a major QTL centered on the *D* locus, is largely responsible for white, yellow, orange and red wing pattern variation (Figure 5C and Figure 4B).

The *D* locus has previously been described as a complex of three tightly linked genes, but recent work strongly suggests that the homeobox transcription factor *optix* is the sole protein-coding element responsible for the activity of *D* [1]. Starting at ∼60 hours after pupation *optix* expression precisely prefigures all *D*-controlled red, orange, and white wing patterns in *H. erato*, its co-mimic *H. melpomene*, and other *Heliconius* species [1]. Interestingly, *optix in situ* hybridization matches perfectly the white and red forewing bands in *H. melpomene plesseni*, which is the co-mimic of *H. e. notabilis*, the species used in our QTL study. Although no *in situ* expression data are currently available for *H. e. notabilis*, we would expect to observe an identical pattern as in *H. m. plesseni* given the strong correlation described between the spatial expression of *optix* in all the *H. erato* and *H. melpomene* wing tissues examined [1]. Overall, the *optix* protein is highly conserved in *Heliconius* and the switch between red/orange and non-red/orange scales is explained by allelic variation of *optix cis-*regulatory elements [1]. The simplest model for how *optix* might regulate color pattern variation imply that different *cis-*regulatory elements of this gene read out different aspects of a conserved species-wide pre-pattern during pupal development. Thus, our results together with observations from previous studies suggest that *optix* acts as a selector gene to specify non-black fates of scale precursor cells where it is expressed.

Our study, however, cannot rule out that the large effect of chromosome 18 may fractionate into several QTLs controlling quantitative variation in pattern color composition independently of *optix*. The resolution of a QTL-mapping study depends on the number of offspring and the size of a QTL effects [40]–[42]. Thus, the combination of the small sample size of this study (88 offspring) and the large effect size of the *optix* gene restricts our ability to distinguish between a single QTL and a series of tightly linked QTLs [41], [42]. The quantitative variation explained by only the *D/optix* locus on chromosome 18 under the additive model is significantly large with over 60% for the amount of red and roughly 85% for the proportion of non-red scales that are either white or yellow (Table 1). In spite of these limitations, our QTL study clearly suggest that the *D* and *Wh* loci, previously speculated to be unlinked in *H. erato* [11], are actually mapping to a the same region of LG 18. Interestingly, the yellow/white switch in *H. cydno* is modulated by a different locus, which is linked to the *wingless* gene [17] and maps our LG 1 (Figure 4). There is no evidence that this linkage group explains any of the variation in white and yellow observed in our crosses, which is nearly completely explained by the large effect QTL in chromosome 18 and a sex-linked QTL (Figure 4B and Table 1). Thus, different *Heliconius* species have evolved the ability to modulate the shift between yellow (3-OHK pigment) and white (no pigment) scales cells in at least two distinct ways.

Despite the major control of the *D*/*optix* locus on qualitative and quantitative color variation, other regions of the genome play an important role in defining *H, erato* wing color pattern. Of particular significance is the presence of three QTLs on three different chromosomes. These loci explained nearly 30% of residual forewing variation in the amount of red, which varied from nearly all red to nearly all white/yellow under the epistatic model (Figure 3; Table S5). Two of these QTLs, on LG 5 and LG 4 respectively, accounted for the bulk of this additional variation and each of them interact epistatically with *D* to reduce the amount of red across the forewing band. As mentioned above, these results are consistent with a recent study demonstrating that *optix* is expressed in the developing wings of *H. m. plesseni* (i.e. the comimic of *H. e. notabilis*) with a spatial distribution that prefigure the red/white upper and lower forewing bands. Thus, while *optix* acts as a major determinant of non-black wing scale identity, additional modifiers interact with the pathway regulated by *optix*. This implies that there are additional loci that repress pigment synthesis in order to temper the final color fate. We speculate these color-repressing modifiers may modulate the expression of transporters or enzymes required for ommochrome pigment synthesis.

### Forewing band variation in *Heliconius erato*

Our crossing experiments included races that represent most of the diversity in forewing band size and shape within *H. erato*. Sheppard and colleagues [11] had previously assigned differences in forewing band shape variation in *H. erato* to a number of distinct loci (Figure 2). However, similar to the red locus, integrative mapping experiments confirm that a single genomic interval, and possibly a single locus centered on *Sd*, is responsible for much of the adaptive variation in forewing band shape, size and position (Figure 6). As discussed for the QTL centered on the *D* locus, also in this case we cannot exclude the possibility of multiple tightly linked loci around *Sd*. In particular, for the relative size of the upper forewing band (NS) there is a peak in LOD scores at *Sd* followed by a curious step rise in values towards the end of the same chromosome (Figure 6). One interpretation of this pattern is that at least two QTLs on LG 10 account for the observed variation: the one centered on *Sd* has an effect on both mid and upper forewing patches whereas the second QTL, at the end of the chromosome effects uniquely the size of the upper forewing band (Figure 6 and Figure 7). As observed for the yellow, red, and white color pattern variation it is likely that the developmental toolkit for altering melanic patterns on *Heliconius* wings involves the activity of additional loci. At least seven different QTLs across five different linkage groups collectively accounted for differences in the distribution of melanic black pattern elements across the upper and lower forewing.

Recently, high resolution linkage mapping, expression analysis and pharmacological experiments unambiguously suggest the *Heliconius* homolog of *WntA* as the *Sd* locus [19]. The spatial pattern of *WntA* expression corresponds to the black pattern [19] and suggests that *WntA/Sd* controls the black “shutter” effect produced by pattern expansion from the organizing centers (Fig. 7). Similarly to *optix*, variation in the *cis* regulatory region of *WntA* is responsible for the adaptive evolution of black forewing patterns in *H. erato* [19]. *WntA* is a signaling ligand that creates a morphogen gradient across the developing wing tissue, which possibly induce and maintain the expression of different target genes at distinct concentration thresholds. *WntA* is transcribed during last-instar imaginal disc development, although pharmacological manipulations suggest that *WntA* signaling actually occurs shortly after pupation. Thus, *WntA* activity precedes color pattern expression of *optix* by a few days. Such observations, leads us to hypothesize that *WntA* may be a direct or indirect negative regulator of *optix*, and that the interaction between the two genes is largely responsible for establishing black vs. non-black wing pattern boundaries. Moreover, recent transcriptomic work shows that late-pupal expression of some ommochrome-related genes closely corresponds to *optix* expression [43]. Hence, while *WntA* possibly controls a variety of molecules during early development to create pattern boundaries that do not directly activate melanin deposition *optix* acts directly on the ommochrome pathway switch later in development. We speculate that *WntA* and *Optix* interaction could provide the fundamental mechanisms to control the natural diversity observed in *Heliconius* wing color patterns.

*WntA* is exactly the type of molecule predicted by theoretical and developmental models for color pattern formation in butterflies. An important organizing principle of butterfly color patterns is the nymphalid groundplan (NGP), first deduced by Schwanwitsch [44] then integrated and expanded by Nijhout [35]. The NGP proposes that color pattern development is controlled by an array of organizing centers distributed across the wing. Signals from these organizing centers determine the boundaries of the various elements of the color pattern. These organizing centers are distributed in irregular rows that run roughly in an antero-posterior direction across a wing [45]. Among the best-studied of these are the organizing centers for eyespot patterns [46]–[51]. Notch signaling and the expression of distal-less are activated at these centers [46], [52], followed by the expression of spalt and engrailed [48]. Depending on the species, different combinations of these transcriptional regulators determine the black central disk and the outer rings of an eyespot [48], [52], [53].

The bold black regions on the wings of *Heliconius* and *Charaxes* can be shown to come about by the confluence of smaller black pattern elements that expand from organizing centers that are homologous to those that organize eyespot patterns [25], [26], [54]. The locations of these organizing centers, and the manner in which they expand and fuse to form large areas of black pigmentation, are presented in Figure 7. The rows of organizing centers in *Heliconius* wings are labeled c, d, f and h in Fig. 7A, were each letter represents a set of serially homologous centers corresponding to elements of the NGP [25], [35], [55]. Some of the genes that control color patterns in *H. erato* and *H. melpomene* have been shown to affect the degree of expansion of these black areas [26]. Gilbert [27] referred to these genes as “shutter genes,” an elegant metaphor that describes how they cause black portions of the pattern to expand and cover up the more brightly pigmented background. This groundplan provides an explanation of the developmental mechanism responsible for the phenotypic effects of the *WntA*/*Sd* gene (Fig. 7B). Each of the *WntA*/*Sd* alleles appears to affect the degree of distal expansion of black pattern from one (f) or two (d and f) of the serially homologous organizing centers in either the anterior compartment of the wing (anterior to vein M_3_ [45]), or in both compartments. Thus each allele has a characteristic effect on the same subset of organizing centers. This action is entirely driven by changes cis-regulation of *WntA* [19], which effects in which location of the wing *WntA* expression occurs. Finally, it is noteworthy to mention that there are several additional *Wnt* family members on the same chromosome (LG 10) nearby *WntA* that might explain the two distinctive QTL peaks (*Sd/WntA* and possibly a color pattern gene named *Ro*) that we observe in our analysis on such chromosome. These loci become interesting candidates for future developmental analysis.

Comparative mapping and expression experiments suggest that the *Sd/WntA* locus is homologous to the *Ac* locus in *H. cydno* [17], [19]. *Ac* controls the presence/absence of melanic scales on central portions of the forewing and hindwing in *H. cydno* [17]. Similar to *H. erato*, several loci in *H. melpomene* and *H. cydno* have been proposed to be responsible for variation in melanin patterns across the forewing and hindwing of different color pattern races of each species. For example, distinct studies list several different loci that have some effect on the shape of the forewing banding patterns in *H. melpomene* [11], [26], [35] and *H. cydno* [26], [35]. Similarly, our expectation for *H. cydno* and *H. melpomene* is that the number of unique loci involved in black patterning within each adaptive radiation will collapse to a much smaller set of loci, each containing large effect alleles, similar to what we observe in *H. erato*.

### The genetic architecture of adaptation and speciation

By taking advantage of a wide range of crosses generated with distinct *H. erato* morphs, our study describes the genetic architecture underlying quantitative wing color pattern variation in *H. erato* at greater detail than has been previously possible. Our work expands upon two previous studies [56], [57] that measured quantitative wing color pattern variation in *Heliconius* butterflies in order to increase the overall knowledge on the genetic control of this diversity. Although the QTL analysis from in *H. melpomene* [56] and *H. numata* [57] used relatively few markers and were largely restricted to chromosome level analysis, their main conclusions are very similar to ours (Table 2). The broadly interpretation of these previous studies and ours is consistent with the presence of a small conserved set of major effect genes that interact with a larger set of “modifiers” in order to modulate wing pattern variation in *Heliconius* butterflies. For example, Baxter and collaborators [56] identified six chromosomes significantly affecting the size variation of the red forewing band in *H. melpomene* (Table 2). Among these chromosomes with significant QTLs in *H. melpomene*, the ones for which we can confidently identify homology with *H. erato* are LG 18 (*D*) and LG 10 (*Sd*) and sex Z chromosome (Table 2). The second quantitative assessment of color pattern in *Heliconius* wings [57] focused on the variation observed in two broods of *H. numata*; a species where a single supergene named *Pushmipullyu* (*P*) [58] control almost entirely its natural wing pattern diversity. Unexpectedly, this study was able to demonstrate that other genomic regions beside the *P* locus, contribute to the overall variation observed in the *H. numata* wing patterns (Table 2). Similarly to the QTL analysis in *H. melpomene* [56], Jones and collaborators [57] found that the regions homologous to the *D* and *Sd* loci of *H. erato* had a significant effect on *H. numata* quantitative wing pattern variation (Table 2). Overall our study adds another level to the analysis, and somewhat paradoxically while simultaneously demonstrating that many previously described gene-loci collapse into alternate alleles of a single locus we have identified new QTL's on other chromosomes that modify the effect of the major wing patterning loci in these species.

**Table 2:** Summary of chromosomes with QTLs in this study and overall comparison with previous works.

	*QTLs IN THIS STUDY*	*QTLs ACROSS STUDIES USING H. melpomene AS REFERENCE*			
Study	This study	The HCG (2012). Reference genome.	This study.	Baxter *et al.* (2006).	Jones *et al.* (2011).
Cross information	*H. erato: notabilis×himera*	*H. melpomene: rosina×melpomene*	*H. erato: notabilis×himera*	*H. melpomene: melpomene×cythera*	*H. numata: elegans het.×aurora het. elegans het.×silvana*
**Likage ID**	LG1	Ch1	LG1	LG1	LG1*
	LG2*	Ch2	na	LG2*	LG2
	LG3*	Ch3	na	LG3	LG3
	LG4*	Ch4	na	LG4	LG4
	LG5*	Ch5	na	LG5	LG5
	LG6*	Ch6	na	LG6	LG6
	LG7*	Ch7	na	LG7*	LG7
	LG8	Ch8	na	LG8	LG8
	LG9	Ch9	na	LG9	LG9
	LG10 (Sd_not)*	Ch10 (*Ac*)	LG10 (*Sd*_not)*	LG10 (*Ac*)*	LG10*
	LG11	Ch11	na	LG11	LG11
	LG12*	Ch12	na	LG12	LG12
	LG13	Ch13	na	LG13*	LG13
	LG14	Ch14	na	LG14	LG14
	LG15 (*Cr_*not)	Ch15 (*Yb*)	LG15 (*Cr*_not)	LG15	LG15*
	LG16	Ch16	na	LG16	LG16
	LG17	Ch17	na	LG17	LG17
	LG18*	Ch18 (*B/D*)	LG18 (*D*)*	LG18 (*B/D*)*	LG18*
	LG19	Ch19	na	LG19	LG19*
	LG20	Ch20	na	LG20	LG20
	LGZF*	Ch21	LGZF*	LG21*	LG21*

In the grey column on the left of the table the linkage groups with at least one QTL locus identified from our study are indicated with an asterisk. In the rest of the table in white, the comparison between this work and previous studies are summarized using *H. melpomene* linkages numbers as a reference (NA indicates the linkage groups where homology could not be established).

In a broader context, the overall genetic architecture of *Heliconius* color pattern variation described in this study supports the two-step process of Müllerian mimicry evolution, as proposed by Punnett [59], elaborated by Nicholson [60], and finally presented with a more complete heuristic view by Turner [61]–[63]. This model entails that the first large effect mutation allows the future mimic to cross a fitness valley from a lower adaptive peak to a higher adaptive slope determined by a warningly colored model species. Then, smaller changes will permit climbing, over time, to the highest adaptive peak imposed by the model and generate a perfect mimetic resemblance. Turner adopted this two-step model to explain how pattern evolution in *Heliconius* butterflies would occur under the selective regime imposed by vertebrate predators. Turner [64], [65] and others [39], [60] recognized that only a finite number of adaptive peaks exist in nature, and that these peaks in *Heliconius* butterflies are easily visualized as distinctive Müllerian mimicry rings (i.e. groups of distantly related species that share a common warning signal). Each of these rings can contain a large number of distantly related species but only a handful of distinctive rings coexist in any given location [63]. Thus, Turner proposed that mutations of large effect are required to move individuals across phenotypic space between existing mimicry rings. In order to be fixed by selection, such mutations need to be capable of generating a pattern that falls under the protection of an existing mimetic pattern. Then, once a phenotypic shift has occurred, mutations of smaller effect can hone the pattern and perfect mimicry [62], [66]. Several theoretical models have also recently been described in favor of a multi-peaks adaptive landscapes [67]–[70] and field experiment that demonstrated adaptive peaks with valley between them [13]. Moreover, there is a long history field experiments in *Heliconius* that show strong selection against oddly patterned individuals [13], [71], [72] necessitating initial steps of mimetic resemblance to be ‘large’ and generate a ‘rough & ready’ appearance for any hope of mimetic phenotypes to evolve.

As a final remark, our study provides additional data and insights into the genetic mechanisms driving mimicry evolution and more in general the genetic architecture of adaptive variation. The central debate on mimicry evolution has always focused on the number of steps (few versus many) and their effect size (small versus large) needed in order to resemble the model. Overall, our data are consistent with recent theoretical work modeling the distribution of effect sizes over the course of an adaptive walk towards a local optimum [66], [73]. One of the most robust conclusions from these models is that only a few mutations are expected to account for most phenotypic change. Also, the overall size effect of the mutations that are substituted over entire bouts of adaptation fits an exponential distribution. Such a distribution implies that while the first mutations have large phenotypic effects the subsequent ones decrease exponentially their effects as they accumulate. Under this prediction a population should take few larger steps when far from the optimum, but many small ones when near. A number of empirical studies have been borne out of the above expectations [3], [74]–[77]. This new theoretical understanding coupled with emerging empirical examples underscore a broad conceptual change in our understanding of the genetic basis of adaptation since Fisher's first models over 70 years ago [66], [78]. In particular, one of Fisher's main arguments, is that mutations of small phenotypic effect are more likely to be beneficial than are those of large phenotypic effect. What our data suggests, is that in wing mimicry systems small effect mutations are used to optimize only the characteristics of the phenotype controlled by a major effect gene that needs improvement while preserving, or slightly effecting, the rest. Ultimately, our comprehension of how adaptive novelty arises will become more robust as we integrate the developmental events underpinning the formation of specific morphologies with evolutionary and genetic theory (sensu Stern) [79].

## Materials and Methods

### Sample collection and crossing strategy

We generated F_2_ and backcross mapping families by crossing four different geographic races of *H. erato* to the same stock of *H. himera* (Figure 1). Races included: 1) *H. erato notabilis* (north-eastern Ecuador), 2) *H. erato etylus* (South-Eastern Ecuador), 3) *H. erato cyrbia* (western Ecuador), and 4) *H. erato erato* (French Guiana). This design allowed us to score *H. erato* color patterns against the common phenotypic background of *H. himera*, which has a fully developed middle forewing yellow band and a red hindwing bar. All crosses were carried out in the *Heliconius* Insectaries at the University of Puerto Rico from stocks originally collected in the wild. Individuals used to established our stocks were collected from the following locations: *H. himera*, Vilcabamba, Ecuador (79.13 W, 4.6 S); *H. e. notabalis*, Puyo, Ecuador (78.0 W, 1.5 S); *H. e. cyrbia*, Guayquichuma Glen, Ecuador (79.6 W, 3.9 S); *H. e. etylus*, Zamora River, Ecuador (78.5 W, 3.55 S); *H. e. erato*, Maripasoula, French Guiana (54.03 W, 3.64 N). Butterflies were cared for as outlined in [80]. Offspring in all mapping families were reared individually and raised until eclosion. Adult butterflies were uniquely identified with a number, their bodies stored at −70°C for genetic analysis, and high-quality standardized pictures of their wings taken with a Nikon Coolpix 995 digital camera and a single Vivitar 2400M external slave flash mounted on a custom arm held at a 30 degree angle with respect to the plane of focus. The camera was fixed in a custom-built copy stand, using constant illumination and camera settings (F10, 1/2000^th^ shutter speed, 20.2 mm focal length, constant distance from subject and a neutral gray background overlayed with a with 5 mm grid, in sRGB color space) for further morphological analysis.

### Scoring wing pattern phenotypes

Genotypes for the color pattern genes segregating in our crosses were scored based on qualitative measures [12], [20]. Although qualitative scoring does not capture all of the segregating variation (Figures 1 and Figure 3), most color pattern difference within individual crosses can be explained by simple allelic variation at a handful of loci. We scored our broods for a total of six previously named color pattern loci (*Sd*, *St*, *Ly*, *D*, *Cr*, and *Wh*) that segregated in our collection of crosses (see Figure 1 and Figure 2). A short description of the action of these genes, based on the work of Sheppard and collaborators [11] and elaborated in several studies [81], [20], [22] is illustrated in Figure 2 and described below.

### Action of major color pattern genes

The color pattern variation segregating in our crosses has been previously ascribed to the following hypothesized loci: *D*, *Wh*, *Sd*, and *St*, in *H. e. notabilis*×*H. himera*; *D* and *Sd* in *H. e. etylus*, ×*H. himera*; *D* and *Ly* in *H. e. erato*×*H. himera*; *D* and *Cr* in *H. e. cyrbia*×*H. himera* (Figure 1 and Figure 2). Brief descriptions of the major phenotypic action of each locus are as follows:

*D* (modified from *DRY*): This locus controls red color patterns in *H. erato* and was originally proposed by Sheppard *et al.* [11] to be a “supergene” of three tightly linked loci *D(ennis), R(ays), and Y(ellow band)*. *D* controls the red/orange at the base of the forewing; *R* organizes the red/orange rays in the hindwings; and *Y* controls the color of the forewing band, which can be yellow or red. Recently, we presented strong genetic and gene expression evidence that the three loci are cis-regulatory elements of the transcription factor *optix* [1]. For simplicity and, in keeping with more recent nomenclature, we refer to the *DRY* locus as *D*.

*Wh* (*White*): This locus controls the abundance of white scales on the forewing band present in the east Ecuadorian race *H. e. notabilis*. Sheppard *et al.* [11] hypothesized an interaction and/or linkage of the *Wh* locus with another color pattern locus, but they were unable to assign the *Wh* locus to a linkage group.

*Sd* (*Short band*) and *St* (*Split band*): Sheppard *et al.* [11] characterized two tightly linked loci, *Sd* and *St*, in crosses that involved the east Ecuadorian race *H. e. notabilis*. *Sd* is responsible for shortening the forewing band relative to other *H. erato* races while the *St* gene controls the splitting of the forewing band into two portions (Figure 2). The name *Sd* was also used to describe the locus controlling forewing band variation in *H. e. etylus*×*H. himera* crosses [22] but without evidence of homology with *Sd* as defined in the original cross.

*Ly* (*Broken band*): The major phenotypic action of this gene is to alter the forewing band shape, causing it to be fractured relative to the solid forewing band of other *H. erato* races. The locus was described from crosses between Suriname hybrids (*H. e. amazona*×*H. e. hydara*) and population from Trinidad (*H. e. hydara*) and was subsequently confirmed by crossing populations of *H. e. amalfreda* from Manaus, Brazil and *H. e. phyllis* from East Brazil [11], [82] (see Figure 2).

*Cr* (*Cream rectangles*): This locus controls a variety of characteristics. It was originally described for its effect of weakening the expression of the forewing yellow line of *H. e. phyllis* and the yellow hindwing bar of *H. e. favorinus* [11], [81]. *Cr* also controls a series of white marks along the edge of forewings and hindwings (*i.e. H. e. cyrbia*) and has a pleiotropic effect on the amount of red pigment in the forewing band [20].

### Characterization of wing pattern-linked markers

We used Amplified Fragment Length Polymorphisms (AFLPs) [83] to identify a set of markers across the *H. erato* genome. Genomic DNA was digested with *Eco*RI and *Mse*I endonucleases and a total of 23 AFLP primer combinations (*Eco*CN/*Mse*CNN) were analyzed across several crosses using previously described methods [22], [23], [84]. Linkage analyses were performed using previously described methods [21], [22], and AFLP markers of interest were cloned and sequenced [15]. Three crosses - *H. himera*×*H. e. etylus*, *H. himera*×*H. e. notabilis*, and *H. himera*×*H. e. erato* - were screened for co-segregating AFLP loci (*i.e.* identical fragments segregating in different crosses) on each linkage group containing a color pattern gene. Co-segregating AFLPs linked to pattern genes were excised from gels, cloned size-verified, and sequenced (Table S3). In addition, we also isolated and sequenced an AFLP band (GCAA303) linked to *Sd^ety^* identified in a previous mapping work using *H. himera*×*H. e. etylus* broods [22]. Using this strategy we identified a number of AFLP markers tightly linked (1–3 cM) to the *Sd^ety^*, *Sd^not^*, *St*, *D*, and *Ly* color pattern genes segregating in our crosses and used them as landmarks for linkage comparison between *H. erato* races. A total of 18 AFLP loci within a 3 centimorgan (cM) window from each targeted color pattern gene were identified (Table S4). To supplement the AFLP markers we also designed PCR primers to score several additional anchor loci: *D23/24, Has1, Thap, GPCR, optix* [1], *VanGogh DNAJ, Gn12, Gn47* [28], *GerTra*, *EIF3b* [14], *Wg*, *RPL3*, *RPL22*, *PTC* [22], and *B303* (See Table S2)

### Linkage analysis and map construction

Linkage analyses were performed as previously described in Kapan *et al.* methods [22]. Briefly, we constructed a linkage map using the three-step process first outlined by Jiggins *et al.* [21] and expanded upon by Kapan *et al.* [22]. Due to a lack of recombination during oogenesis in heterogametic females [30], [31], bands from female-informative (FI) AFLP markers were used to identify linkage groups at LOD 8.0 using JoinMap 3.0 [85]. We then used JoinMap 3.0 to compare each “chromosome print” with back-cross informative (BI) AFLPs, as well as codominant anchor loci with segregation ratios of 1∶1∶1∶1 or 1∶2∶1 (see Kapan *et al.* [22]) using groups that form at LOD 4.0 or higher with each chromosome print. These new groups were then individually examined to verify linkage phase and to identify “forbidden recombinants” [86], which are defined as offspring genotypes that could only appear by crossing-over in females. Presence of forbidden recombinants implies that the grouping is incorrect, unless there are scoring errors. In our analysis we accepted a small amount of scoring error and retained BI AFLP loci with five or fewer forbidden recombinants that have a probability of *p*<0.007 or lower of being unlinked under the hypothesis of no scoring error (see Jiggins *et al.* [21]). Finally we extracted the male-informative (MI) component from the BI AFLP scores (i.e. following only AFLP bands inherited from the father) by “censoring” the genotypes from AFLP bands inherited from the mother [21]. The censored BI markers were combined with MI AFLPs and remaining co-dominant anchor markers were re-coded to show only the male-informative allele. For the sex chromosome Z, male offspring are always positive for BI AFLPs and the recombinant analysis is limited to female offspring segregating male-informative markers on Z [21]. For the final map construction linkage groups were assembled at LOD 3.0 utilizing Joinmap 3.0 followed by the use of Mapmaker 3.0 to generate the most likely order and spacing of markers on a chromosome [87].

### Quantification of wing color and size band patterns

*Heliconius* butterflies wing color pattern is not a perfect arrangement of monochromatic areas but it is the result of the ratio between scales of different colors. Such ratio is what determines the final wing color pattern. We used our most phenotypically variable brood as the reference cross (*H. himera×H. e. notabilis: F2-Not9*), to quantify variation in 1) the amount of red across the central forewing patch, 2) the amount of white versus yellow scales (Figure S2), and the size of the upper and lower forewing bars (Figure S1). The amount of red and white scales was extremely variable depending on the area of the forewing band considered. Wings that appeared purely red had a significant portion of white scales and, vice versa wings that seems all white had many red scales. Thus, to estimate redness of the central forewing patch, we used the program SigmaScan® to calculate the intensity of the blue, green and red emission using the Red Green Blue (RGB) color model across the entire forewing band. Therefore, we measured the amount of red, blue and green pixels and calculated “redness” as red emission divided by blue plus green emission (i.e. redness = red/(blue+green)). This measure provided a reasonably robust quantitative estimate of the amount of red in the whole forewing band. However, the RGB data obtained could not be used to distinguish the relative proportion of white versus yellow scale cells in the forewing band due the limitations of digital photographs to capture differences between yellow and white. To obtain a quantitative measure of the relative proportion of white and yellow scales, we used a spectrophotometer to measure the absorption spectrum (see Figure S2). The measured region was in the anterior distal region of the middle right forewing patch (see Figure S2) and was chosen because red scales were mostly absent and thus did not interfere our ability to quantify “whiteness” (or yellowness) of the scale cells in that region. We used the ratio of absorbance at 380 nm and 700 nm as our quantitative measure of “whiteness” (Figure S2). In order to compare the effects of each quantitative trait locus on the different phenotypic measurements (redness, whiteness, yellowness, Big-Spot and Not-Spot) we report the percentage variance explained for each QTL.

*Heliconius himera* and *H. e. notabilis* differ notably in the shape of the forewing band (see Figure 1 and Figure 2). Specifically, the forewing band of *H. e. notabilis* is split with a small patch of white/red scale cells displaced distally towards the upper margins of the forewing. In addition, the central forewing band of *H. e. notabilis* is smaller than the forewing band of *H. himera*. To characterize variation segregating in our focal F2 family, we scored a series of landmarks on standardized wing photos of each offspring (see above) using the computer program DIGIT (courtesy of H. F. Nijhout). DIGIT generates positional (X,Y) coordinates from points on the image. In total, we scored 58 positions across the wing, including 14 “landmark” coordinates chosen to reflect the positions of the major venation patterns and 44 coordinates that reflected the size and position of the proximal and distal forewing bands (see Figure S1). For our analysis we focused specifically on variation in the relative size of two major forewing patches (Figure S1). The Big-Spot (BS) represented the largest portion of the proximal forewing band and the Not-Spot (NS) represented the bulk of the distal band. Although our method measured the relative size variation at the forewing bands by calculating the area for the two bands in each individual, we argue that this measure represents a close approximation of the forewing band shape as well. Quantitative differences size of the BS and NS were measured as an area of the band standardized by the area of the wing, which could be considered as a biological proxy for shape. The area of each spot was calculated as an area of a polygon, with vertices represented by landmarks (Figure S1).

### QTL analysis of color and size data

All QTL analyses were performed in the R statistical package version 2.13.2 [88]. We tested the association between the molecular markers, color and band size and shape phenotypes using a linear regression model with results extracted using the ANOVA function. Association was tested by a simple one-way ANOVA with phenotypes treated as dependent variables, and markers as independent variables. The effect of the marker was assessed via the F ratio, which is equivalent to a LOD score in single marker analyses [89]. Statistical significance of the LOD score was assessed via 5,000 matrix permutations [90]. Permutations resulted in the generation of a distribution of LOD scores, and based on this distribution, only those associations with actual LOD scores having less than 0.01 probability of being observed by chance were considered significant. This probability corresponded to LOD score cut-off values of 3.8 or higher depending on all the linkage groups analyzed. Single marker analyses results were confirmed in the program QTL Cartographer [91] (data not reported).

Since the order and position of markers are known in each linkage group, adjacent markers associated with a phenotype were assumed to present the same QTL. After identification of QTLs associated with variation in color and forewing band size, we ran another one-way ANOVA with only one representative marker per QTL. Markers were chosen on the basis of having the highest explanatory power (highest LOD value) within the QTL. For each phenotype, the selected markers were analyzed individually and then together assuming additivity between markers in a linear regression model. The amount of phenotypic variance explained by each marker and each model was estimated as the r-squared value of the linear regression. We then tested if an additive model better explained the phenotypic variation than a model with just one marker (one-way vs. multi-way ANOVA).

The simplest model of genetic architecture assumes that contributions of individual markers to the phenotype are additive (Fisherian-type genetic architecture) [78]. A more complex model allows for epistasis (Wrightian-type genetic architecture) [92], where the relative contribution of each marker to the phenotype could be dependent on the presence of allelic variants at other markers. To test the explanatory power of these two models, we constructed a purely additive model, which included the marker that has the highest explanatory power in that QTL. Since *optix* is such a strong candidate gene for the *D* locus [1], we tested whether the phenotypic action of the known major effect locus impacting red/orange coloration interacted with other unknown QTLs. We thus examined whether or not interactions between *D* and other makers were significant, and if the epistatic model better explained the data than a purely additive model (additive vs. interaction ANOVA). Similarly, for variation in forewing band size we compared a purely additive and a more complex epistatic model using one marker per QTL and building our model around the marker with the highest explanatory power.

## Supporting Information

Figure S1 **Size analysis.** A and B represent the location of landmarks used for quantitative measure of band size variation in the forewing Big-Spot (BS) and Not-Spot (NS) in a simplified butterfly's wing cartoon and real sample respectively. Functions used for calculations of areas were: **WingSpot** = abs((x3*y10−x10*y3+x10*y11−x11*y10+x11*y12−x12*y11+x12*y13−x13*y12+x13*y14−x14*y13+x14*y15−x15*y14+x15*y16−x16*y15+x16*y3−x3*y16)/2). **Big-Spot** = abs((x25*y7−x7*y25+x7*y6−x6*y7+x6*y31−x31*y6+x31*y32−x32*y31+x32*y33−x33*y32+x33*y34−x34*y33+x34*y35−x35*y34+x35*y36−x36*y35+x36*y37−x37*y36+x37*y38−x38*y37+x38*y22−x22*y38+x22*y21−x21*y22+x21*y20−x20*y21+x20*y19−x19*y20+x19*y18−x18*y19+x18*y17−x17*y18+x17*y25−x25*y17)/2). **Not-Spot** = abs((x50*y51−x51*y50+x51*y52−x52*y51+x52*y53−x53*y52+x53*y54−x54*y53+x54*y55−x55*y54+x55*y56−x56*y55+x56*y57−x57*y56+x57*y58−x58*y57+x58*y49−x49*y58+x49*y48−x48*y49+x48*y47−x47*y48+x47*y46−x46*y47+x46*y45−x45*y46+x45*y44−x44*y45+x44*y43−x43*y44+x43*y42−x42*y43+x42*y41−x41*y42+x41*y50−x50*y41)/2). In the above functions x and y correspond to X and Y coordinates and numbers correspond to landmark numbers. The size of the Big-Spot and the Not-Spot was standardized by the size of the WingSpot.
(TIF)

Figure S2 **Color spectrum and absorbance.** A) Area of the wing measured by a spectrophotometer to distinguish between the amount of white and yellow pigment. B) From right to left the typical absorbance spectrums for individuals that were fully white, yellow, or red is presented.
(TIF)

Table S1 Co-dominant markers in the *D and Cr* interval. Recombinant information for eight loci in the *D* interval and one in the *Cr* interval screened in our collection of crosses are reported.
(JPG)

Table S2 Co-dominant and gene based markers. Information of co-dominant and gene based markers used respectively for linkage analysis and fine scale mapping of the *D* color pattern gene interval. Primers sequence and reference article in which the loci have been developed is reported together with locus ID and linkage information.
(JPG)

Table S3 Anchors AFLP loci. Anchors AFLP loci. AFLP markers isolated and sequenced across our collection of crosses and their relative nucleotide composition are shown. Provenance of allele and characteristic (MI = Male Informative; FI = Female Informative; BI = Both Informative parents) is reported.
(JPG)

Table S4 Tightly linked AFLP loci. AFLP markers tightly linked (≤3 recombinants) to four different color pattern genes (*D*, *Sd^not^*, *Sd^ety^*, *Ly*), are reported. Some of these markers, identified with a star (*) did not enter the final linkage analysis given the very stringent parameters used to create the reference map.
(JPG)

Table S5 Overall QTL analysis for amount of red. QTL analysis for amount of red scales showing chromosomes on which QTLs were found. Only those QTLs with probabilities smaller than 0.01 of occurring by chance were considered significant, and were included in additional analyses. LG = linkage group; marker = marker name; Redness.F.value = LOD score; Redness.Pr.F = probability of observing the LOD score by chance; Redness.c950 = expected LOD score with a P = 0.05 generated via non-parameteric bootstrap; Redness.c990 = expected LOD score with a P = 0.01 generated via non-parameteric bootstrap; Redness.c999 = expected LOD score with a P = 0.001 generated via non-parameteric bootstrap; last three columns = visual representation of marker significance at the P = 0.05 (*), P = 0.01 (**), and P = 0.001 (***) levels.
(JPG)

Table S6 Overall QTL analysis for whiteness. Overall QTL analysis for whitness showing chromosomes on which QTLs were found. Only those QTLs with probabilities smaller than 0.01 of occurring by chance were considered significant, and were included in additional analyses. LG = linkage group; marker = marker name; Whiteness.F.value = LOD score; Whiteness.Pr.F = probability of observing the LOD score by chance; Whiteness.c950 = expected LOD score with a P = 0.05 generated via non-parameteric bootstrap; Whiteness.c990 = expected LOD score with a P = 0.01 generated via non-parameteric bootstrap; Whiteness.c999 = expected LOD score with a P = 0.001 generated via non-parameteric bootstrap; last three columns = visual representation of marker significance at the P = 0.05 (*), P = 0.01 (**), and P = 0.001 (***) levels.
(JPG)

Table S7 Overall QTL analysis for the forewing Big-Spot (BS) size variation. Overall QTL analysis for shape in the forewing Big-Spot (BS) showing chromosomes on which QTLs were found. Only those QTLs with probabilities smaller than 0.01 of occurring by chance were considered significant, and were included in additional analyses. LG = linkage group; marker = marker name; BS Spot,F.value = LOD score; BS Spot.Pr.F = probability of observing the LOD score by chance; BS Spot.c950 = expected LOD score with a P = 0.05 generated via non-parameteric bootstrap; BS Spot.c990 = expected LOD score with a P = 0.01 generated via non-parameteric bootstrap; BS Spot.c999 = expected LOD score with a P = 0.001 generated via non-parameteric bootstrap; last three columns = visual representation of marker significance at the P = 0.05 (*), P = 0.01 (**), and P = 0.001 (***) levels.
(JPG)

Table S8 Overall QTL analysis for the forewing Notabilis Spot (NS) size variation. Overall QTL analysis for size in the forewing Not-Spot (NS) showing chromosomes on which QTLs were found. Only those QTLs with probabilities smaller than 0.01 of occurring by chance were considered significant, and were included in additional analyses. LG = linkage group; marker = marker name; NS Spot,F.value = LOD score; NS Spot.Pr.F = probability of observing the LOD score by chance; NS Spot.c950 = expected LOD score with a P = 0.05 generated via non-parameteric bootstrap; NS Spot.c990 = expected LOD score with a P = 0.01 generated via non-parameteric bootstrap; NS Spot.c999 = expected LOD score with a P = 0.001 generated via non-parameteric bootstrap; last three columns = visual representation of marker significance at the P = 0.05 (*), P = 0.01 (**), and P = 0.001 (***) levels.
(JPG)
