# Supplementary figures and images for: Multi-Allelic Major Effect Genes Interact with Minor Effect QTLs to Control Adaptive Color Pattern Variation in Heliconius erato
Source: PLoS One. 2013 Mar 22;8(3):e57033. doi: 10.1371/journal.pone.0057033 (PMC3606360; doi:10.1371/journal.pone.0057033)

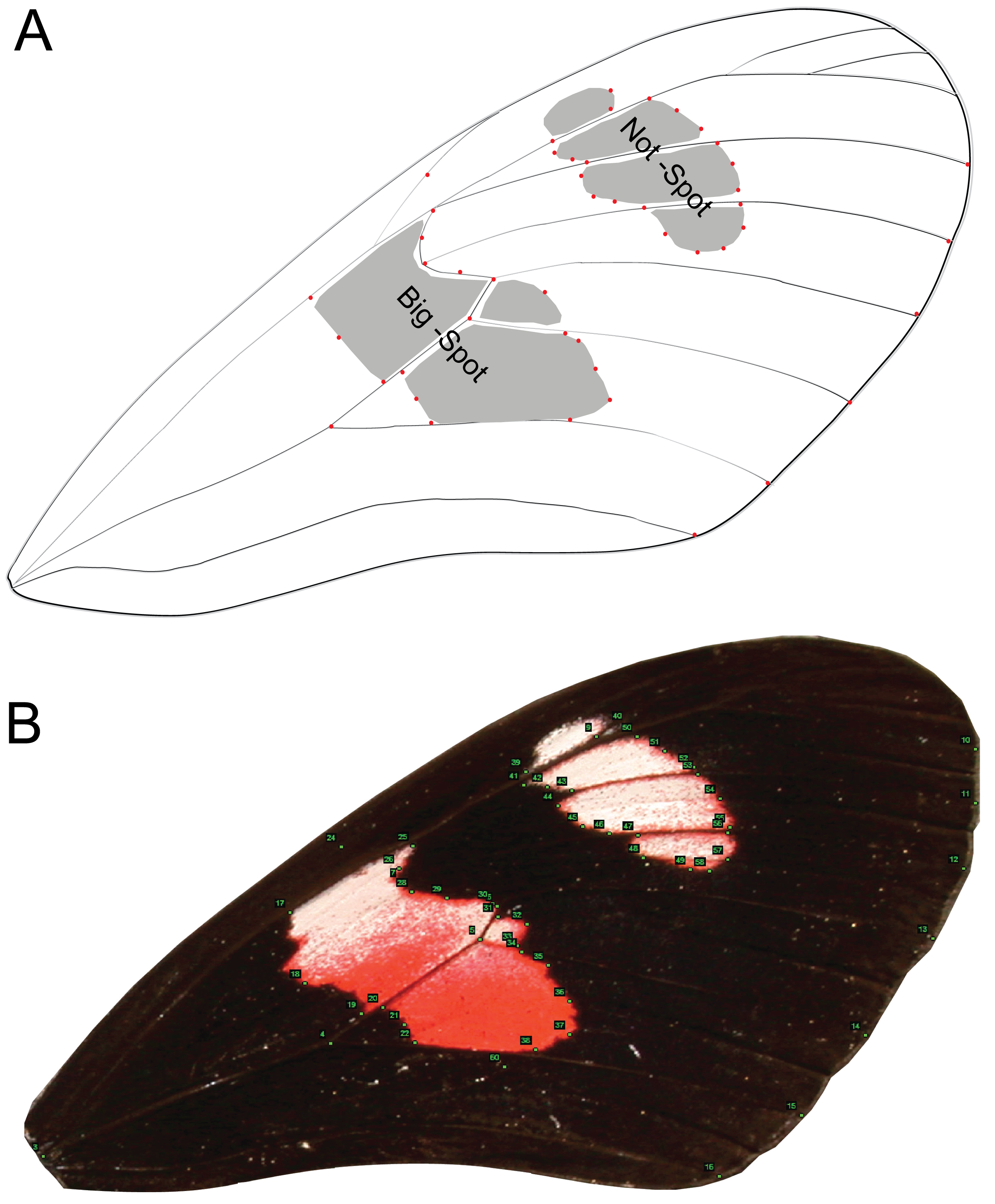

Supplement: Figure S1 — Size analysis. A and B represent the location of landmarks used for quantitative measure of band size variation in the forewing Big-Spot (BS) and Not-Spot (NS) in a simplified butterfly's wing cartoon and real sample respectively. Functions used for calculations of areas were: WingSpot = abs((x3*y10−x10*y3+x10*y11−x11*y10+x11*y12−x12*y11+x12*y13−x13*y12+x13*y14−x14*y13+x14*y15−x15*y14+x15*y16−x16*y15+x16*y3−x3*y16)/2). Big-Spot = abs((x25*y7−x7*y25+x7*y6−x6*y7+x6*y31−x31*y6+x31*y32−x32*y31+x32*y33−x33*y32+x33*y34−x34*y33+x34*y35−x35*y34+x35*y36−x36*y35+x36*y37−x37*y36+x37*y38−x38*y37+x38*y22−x22*y38+x22*y21−x21*y22+x21*y20−x20*y21+x20*y19−x19*y20+x19*y18−x18*y19+x18*y17−x17*y18+x17*y25−x25*y17)/2). Not-Spot = abs((x50*y51−x51*y50+x51*y52−x52*y51+x52*y53−x53*y52+x53*y54−x54*y53+x54*y55−x55*y54+x55*y56−x56*y55+x56*y57−x57*y56+x57*y58−x58*y57+x58*y49−x49*y58+x49*y48−x48*y49+x48*y47−x47*y48+x47*y46−x46*y47+x46*y45−x45*y46+x45*y44−x44*y45+x44*y43−x43*y44+x43*y42−x42*y43+x42*y41−x41*y42+x41*y50−x50*y41)/2). In the above functions x and y correspond to X and Y coordinates and numbers correspond to landmark numbers. The size of the Big-Spot and the Not-Spot was standardized by the size of the WingSpot. (TIF) [file pone.0057033.s001.tif]

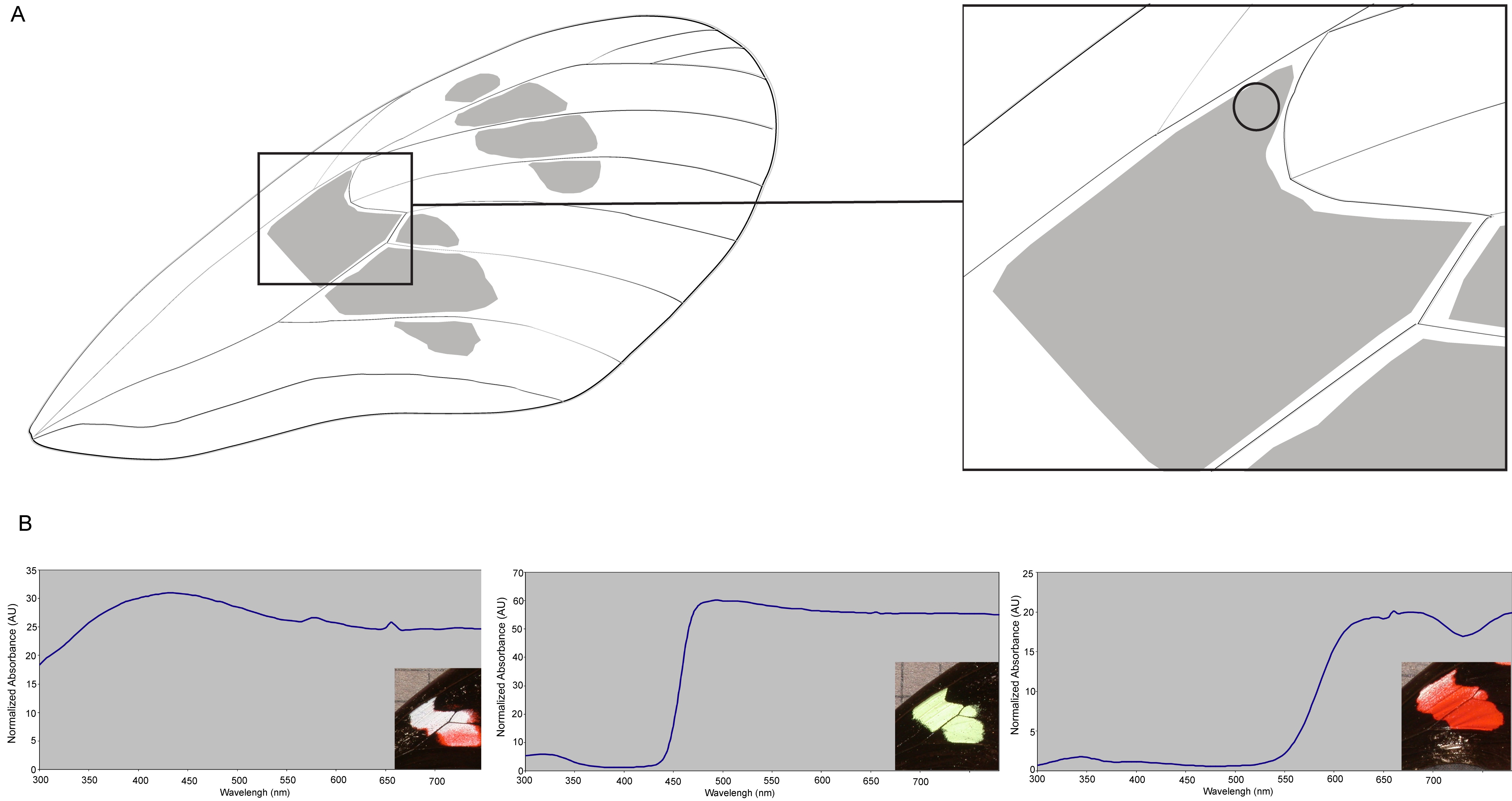

Supplement: Figure S2 — Color spectrum and absorbance. A) Area of the wing measured by a spectrophotometer to distinguish between the amount of white and yellow pigment. B) From right to left the typical absorbance spectrums for individuals that were fully white, yellow, or red is presented. (TIF) [file pone.0057033.s002.tif]

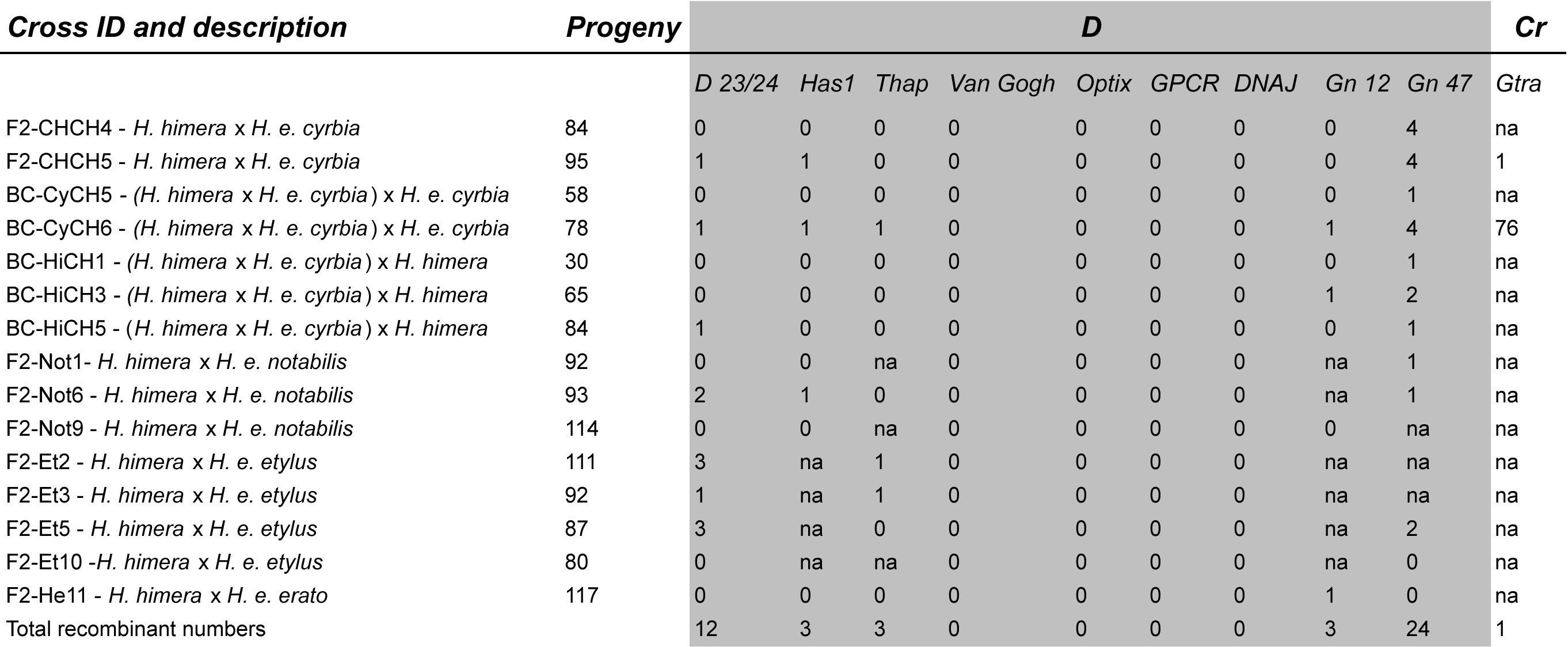

Supplement: Table S1 — Co-dominant markers in the D and Cr interval. Recombinant information for eight loci in the D interval and one in the Cr interval screened in our collection of crosses are reported. (JPG) [file pone.0057033.s003.jpg]

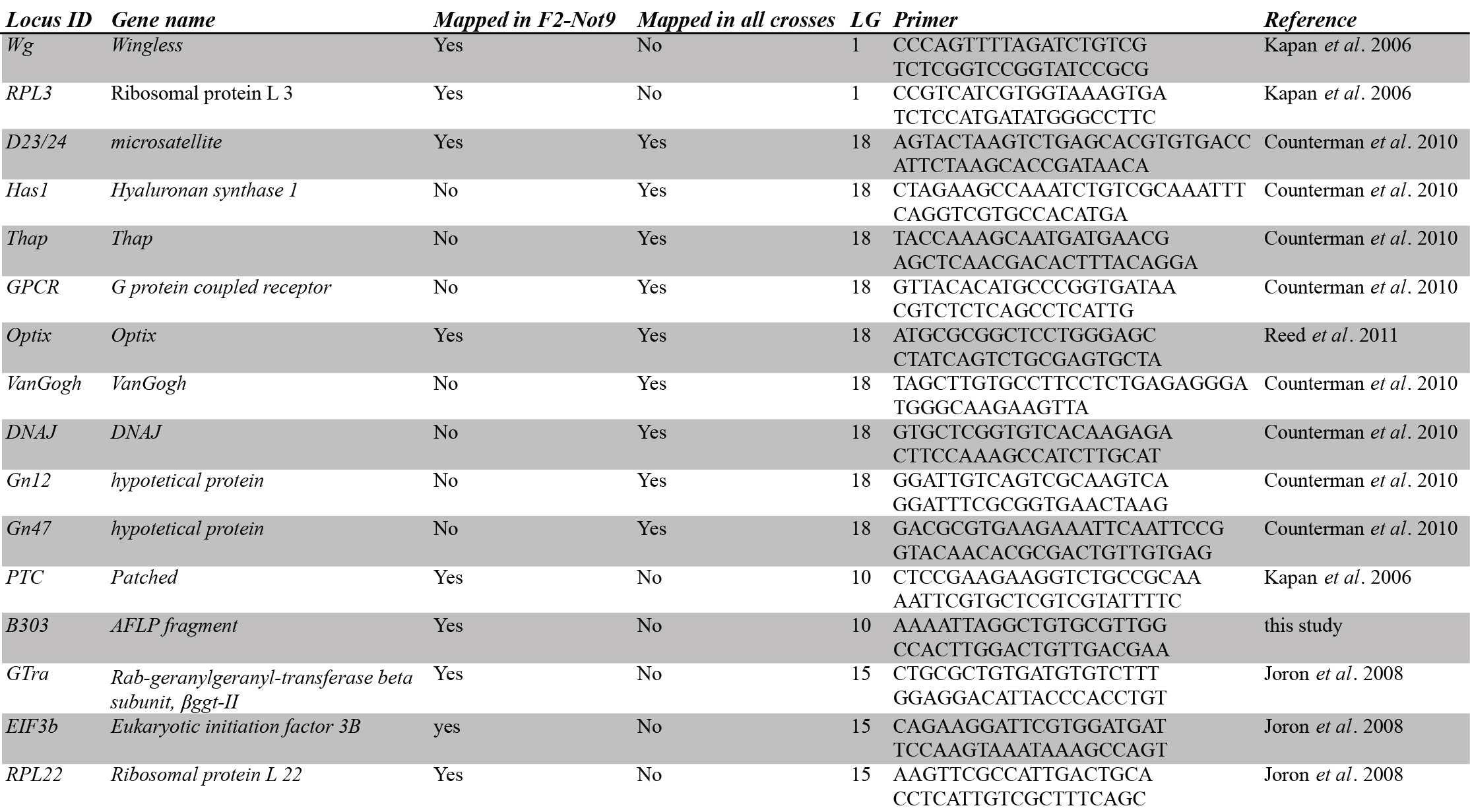

Supplement: Table S2 — Co-dominant and gene based markers. Information of co-dominant and gene based markers used respectively for linkage analysis and fine scale mapping of the D color pattern gene interval. Primers sequence and reference article in which the loci have been developed is reported together with locus ID and linkage information. (JPG) [file pone.0057033.s004.jpg]

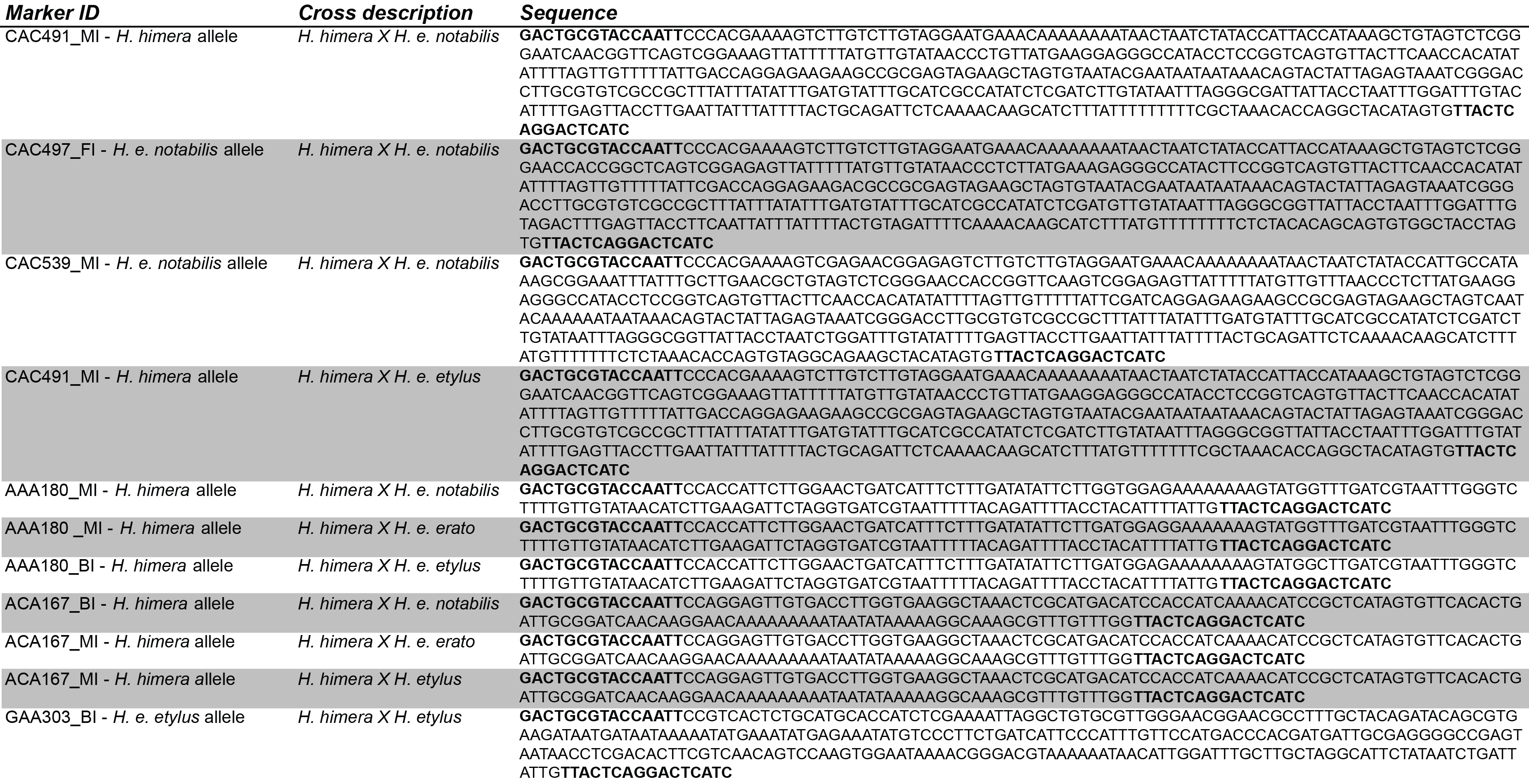

Supplement: Table S3 — Anchors AFLP loci. Anchors AFLP loci. AFLP markers isolated and sequenced across our collection of crosses and their relative nucleotide composition are shown. Provenance of allele and characteristic (MI = Male Informative; FI = Female Informative; BI = Both Informative parents) is reported. (JPG) [file pone.0057033.s005.jpg]

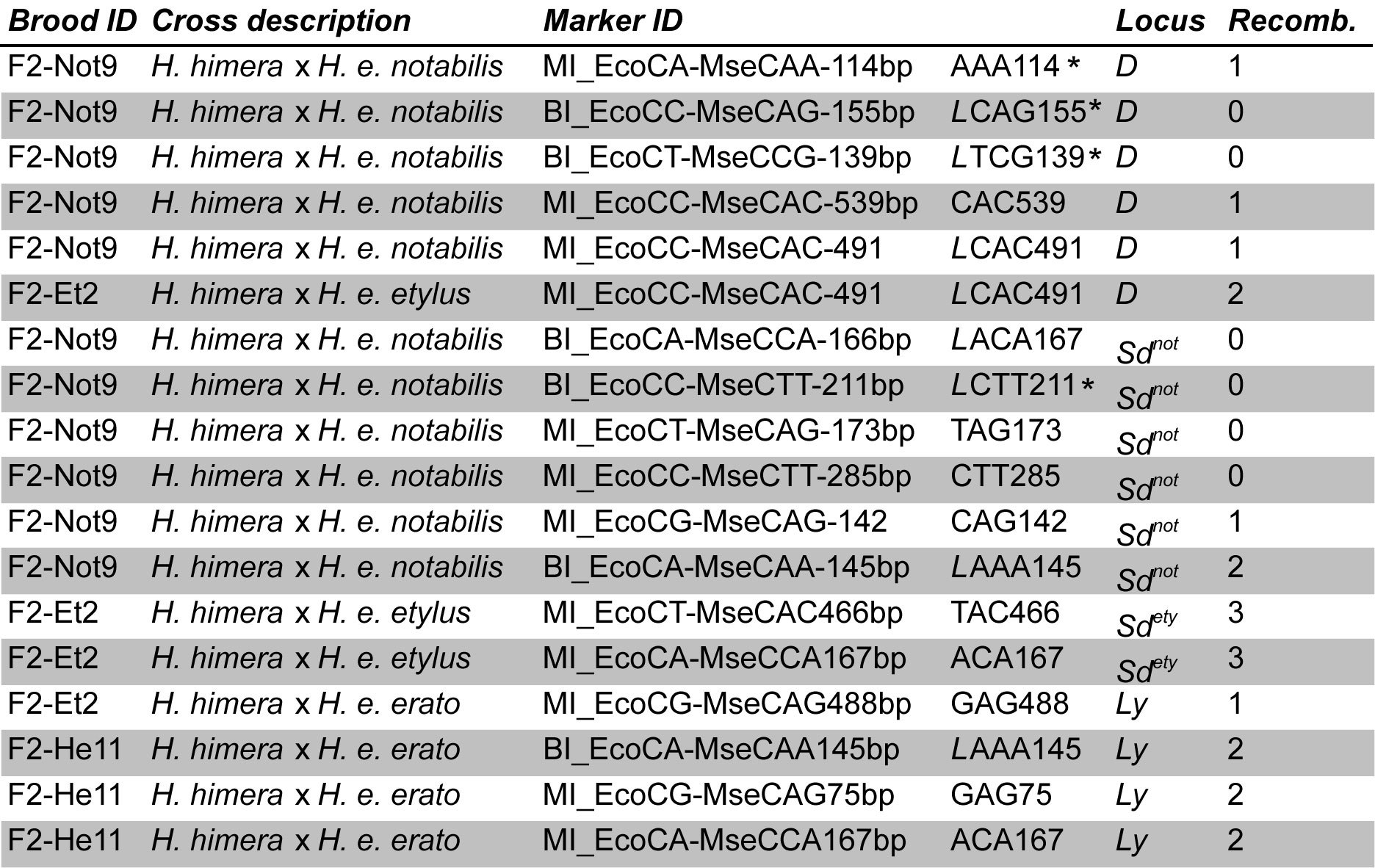

Supplement: Table S4 — Tightly linked AFLP loci. AFLP markers tightly linked (≤3 recombinants) to four different color pattern genes (D, Sdnot, Sdety, Ly), are reported. Some of these markers, identified with a star (*) did not enter the final linkage analysis given the very stringent parameters used to create the reference map. (JPG) [file pone.0057033.s006.jpg]

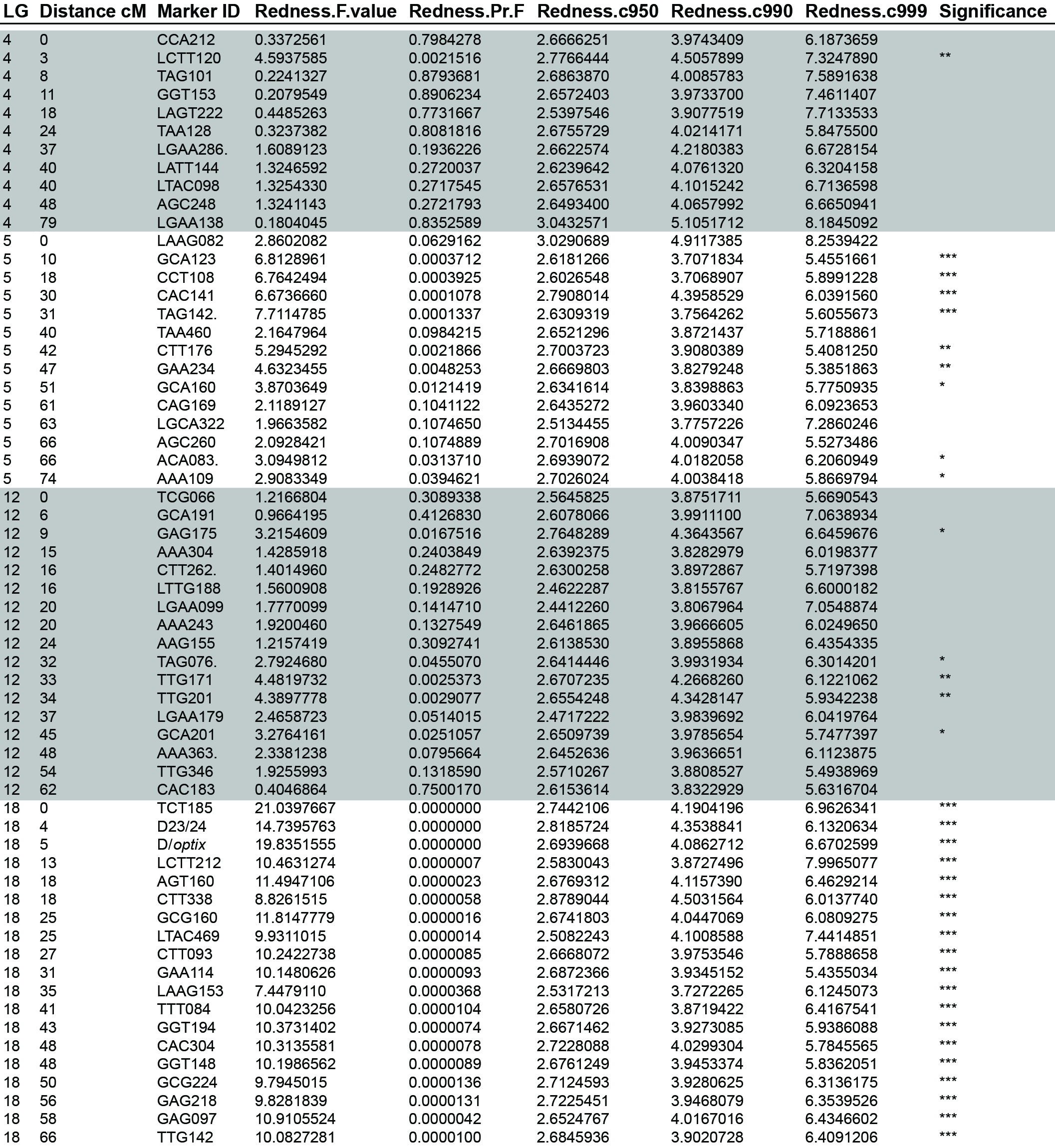

Supplement: Table S5 — Overall QTL analysis for amount of red. QTL analysis for amount of red scales showing chromosomes on which QTLs were found. Only those QTLs with probabilities smaller than 0.01 of occurring by chance were considered significant, and were included in additional analyses. LG = linkage group; marker = marker name; Redness.F.value = LOD score; Redness.Pr.F = probability of observing the LOD score by chance; Redness.c950 = expected LOD score with a P = 0.05 generated via non-parameteric bootstrap; Redness.c990 = expected LOD score with a P = 0.01 generated via non-parameteric bootstrap; Redness.c999 = expected LOD score with a P = 0.001 generated via non-parameteric bootstrap; last three columns = visual representation of marker significance at the P = 0.05 (*), P = 0.01 (**), and P = 0.001 (***) levels. (JPG) [file pone.0057033.s007.jpg]

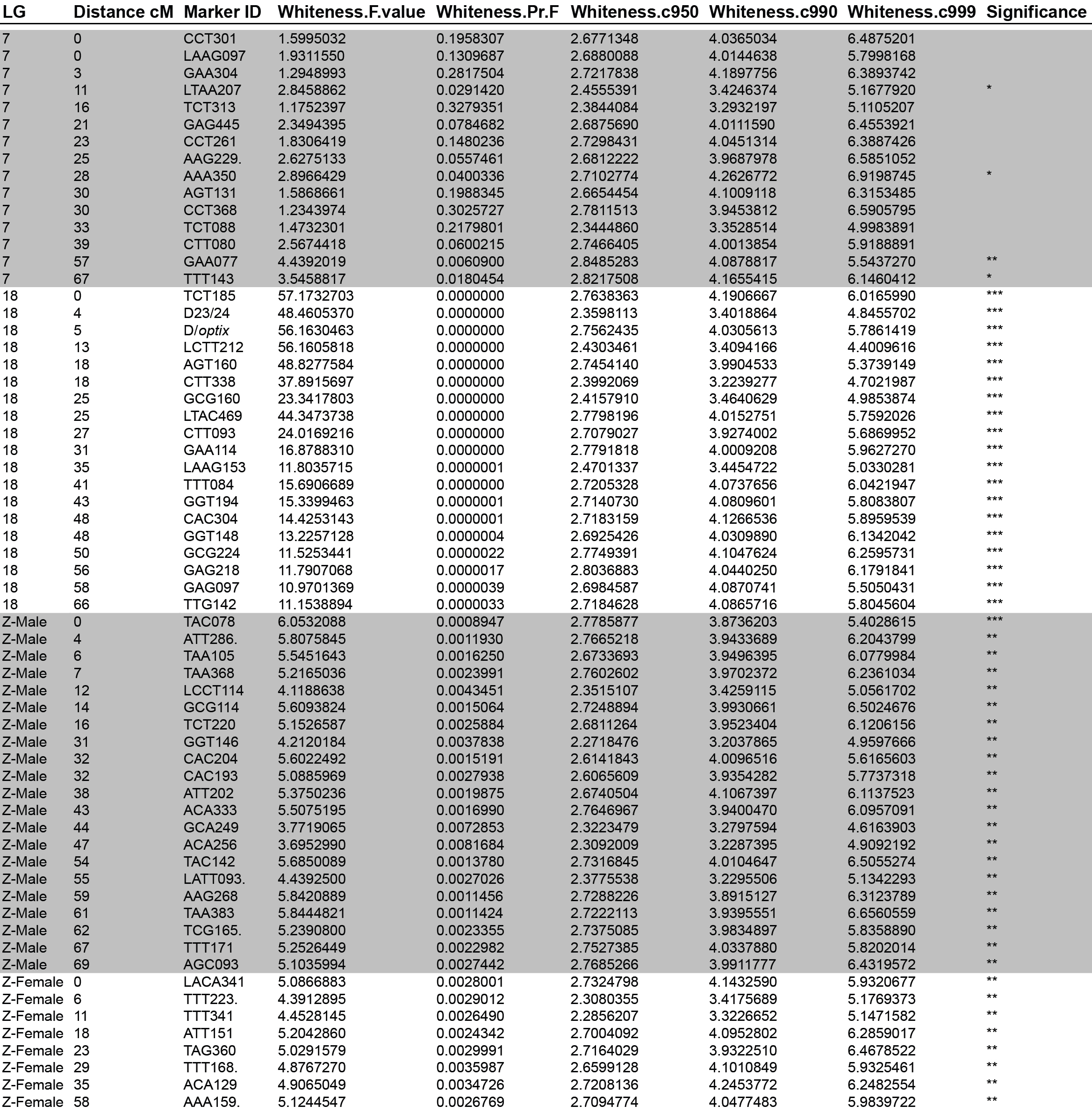

Supplement: Table S6 — Overall QTL analysis for whiteness. Overall QTL analysis for whitness showing chromosomes on which QTLs were found. Only those QTLs with probabilities smaller than 0.01 of occurring by chance were considered significant, and were included in additional analyses. LG = linkage group; marker = marker name; Whiteness.F.value = LOD score; Whiteness.Pr.F = probability of observing the LOD score by chance; Whiteness.c950 = expected LOD score with a P = 0.05 generated via non-parameteric bootstrap; Whiteness.c990 = expected LOD score with a P = 0.01 generated via non-parameteric bootstrap; Whiteness.c999 = expected LOD score with a P = 0.001 generated via non-parameteric bootstrap; last three columns = visual representation of marker significance at the P = 0.05 (*), P = 0.01 (**), and P = 0.001 (***) levels. (JPG) [file pone.0057033.s008.jpg]

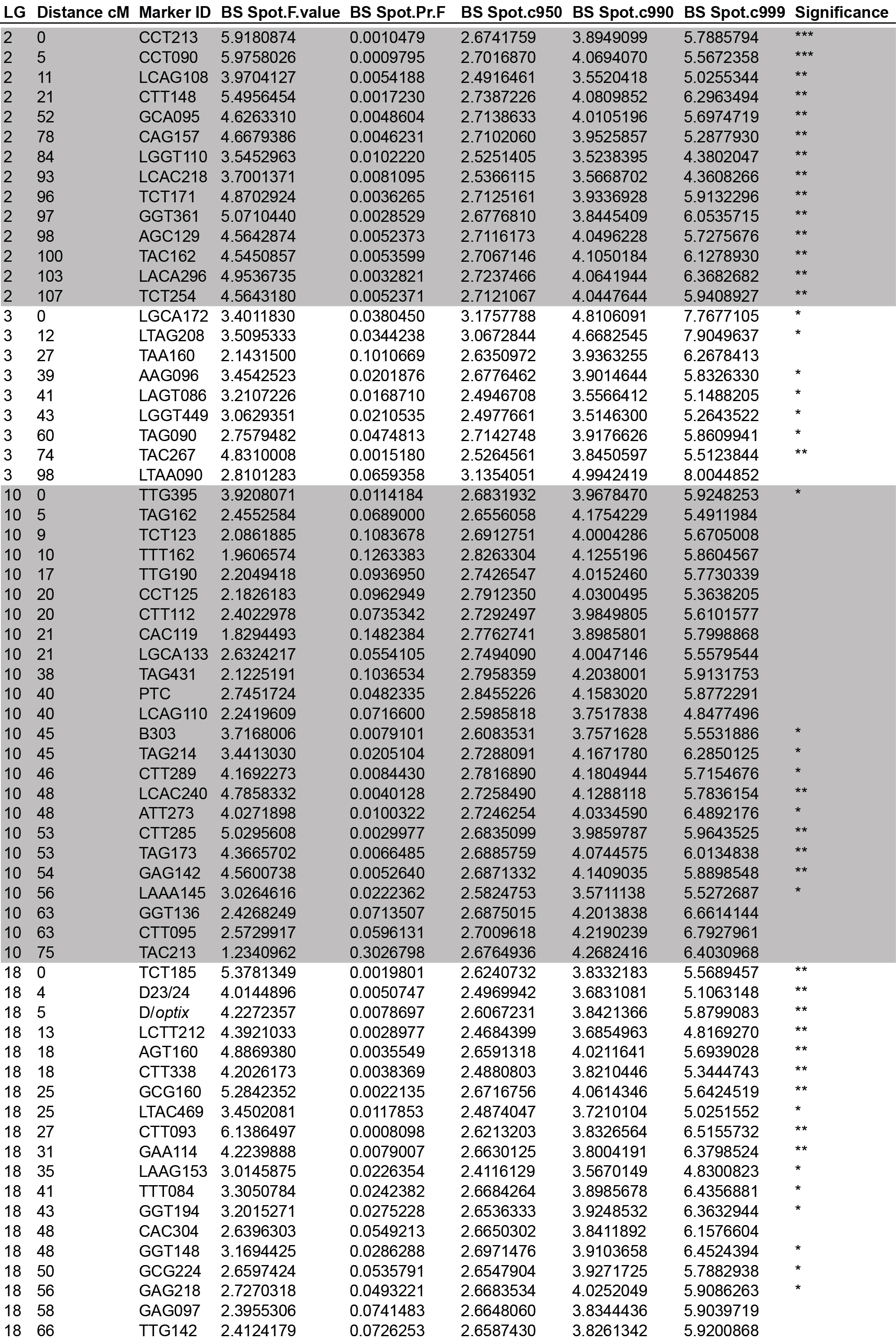

Supplement: Table S7 — Overall QTL analysis for the forewing Big-Spot (BS) size variation. Overall QTL analysis for shape in the forewing Big-Spot (BS) showing chromosomes on which QTLs were found. Only those QTLs with probabilities smaller than 0.01 of occurring by chance were considered significant, and were included in additional analyses. LG = linkage group; marker = marker name; BS Spot,F.value = LOD score; BS Spot.Pr.F = probability of observing the LOD score by chance; BS Spot.c950 = expected LOD score with a P = 0.05 generated via non-parameteric bootstrap; BS Spot.c990 = expected LOD score with a P = 0.01 generated via non-parameteric bootstrap; BS Spot.c999 = expected LOD score with a P = 0.001 generated via non-parameteric bootstrap; last three columns = visual representation of marker significance at the P = 0.05 (*), P = 0.01 (**), and P = 0.001 (***) levels. (JPG) [file pone.0057033.s009.jpg]

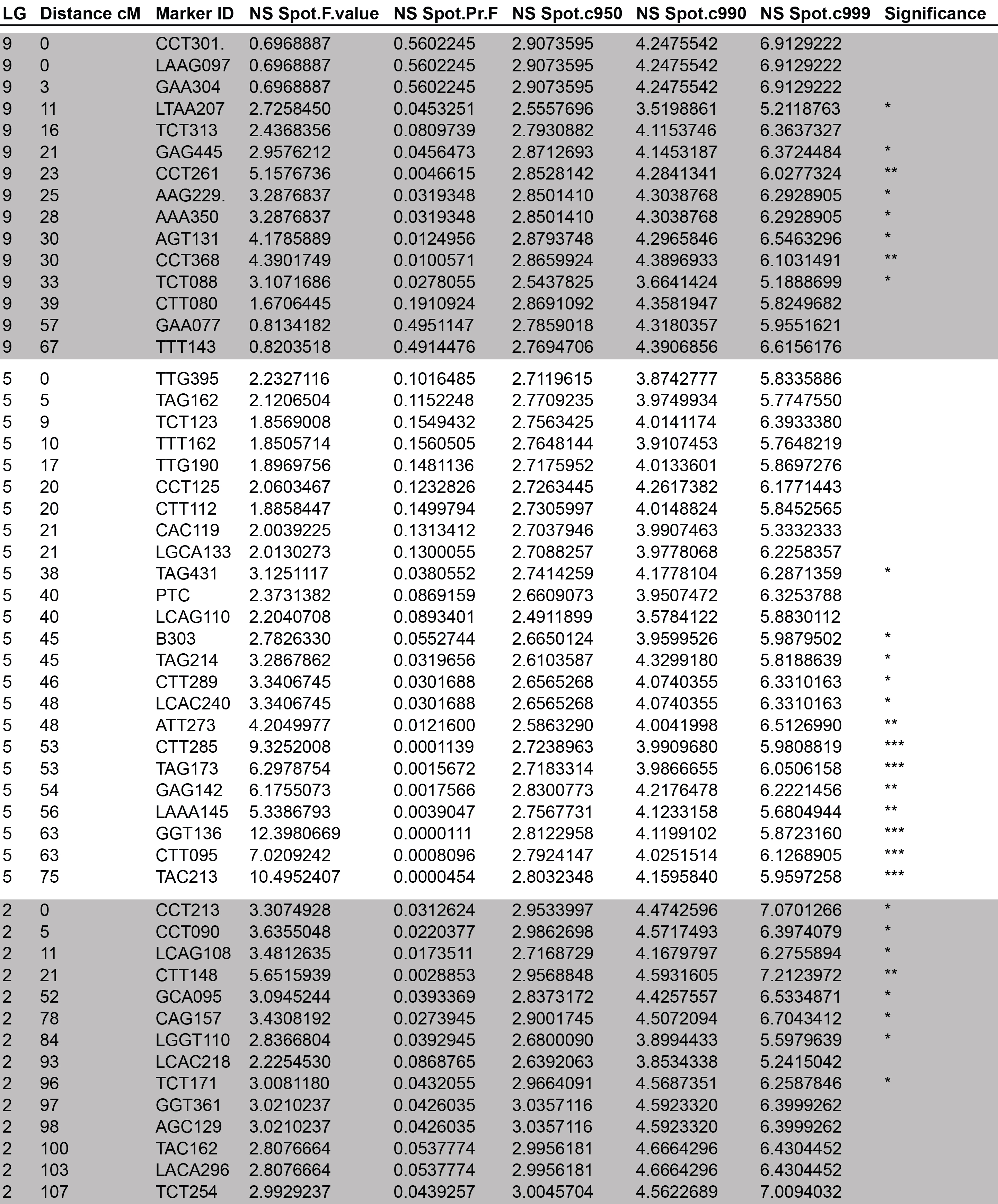

Supplement: Table S8 — Overall QTL analysis for the forewing Notabilis Spot (NS) size variation. Overall QTL analysis for size in the forewing Not-Spot (NS) showing chromosomes on which QTLs were found. Only those QTLs with probabilities smaller than 0.01 of occurring by chance were considered significant, and were included in additional analyses. LG = linkage group; marker = marker name; NS Spot,F.value = LOD score; NS Spot.Pr.F = probability of observing the LOD score by chance; NS Spot.c950 = expected LOD score with a P = 0.05 generated via non-parameteric bootstrap; NS Spot.c990 = expected LOD score with a P = 0.01 generated via non-parameteric bootstrap; NS Spot.c999 = expected LOD score with a P = 0.001 generated via non-parameteric bootstrap; last three columns = visual representation of marker significance at the P = 0.05 (*), P = 0.01 (**), and P = 0.001 (***) levels. (JPG) [file pone.0057033.s010.jpg]
